# ﻿Description of two new genera and seven new species of Plexippina Simon, 1901 (Araneae, Salticidae, Plexippini) from Southwest China, with a review of *Yaginumaella* Prószyński, 1979

**DOI:** 10.3897/zookeys.1257.160127

**Published:** 2025-10-30

**Authors:** Cheng Wang, Ying Wang, Xiaoqi Mi, Shuqiang Li

**Affiliations:** 1 Guizhou Provincial Key Laboratory for Biodiversity Conservation and Utilization in the Fanjing Mountain Region, Tongren University, Tongren, Guizhou 554300, China Tongren University Tongren China; 2 College of Life Science, Shenyang Normal University, Shenyang 110034, Liaoning, China Shenyang Normal University Shenyang China; 3 College of Life Sciences, Anhui Normal University, Wuhu, Anhui 241000, China Anhui Normal University Wuhu China; 4 Anhui Provincial Key Laboratory of Biodiversity Conservation and Ecological Security in the Yangtze River Basin, Wuhu, Anhui 241000, China Anhui Provincial Key Laboratory of Biodiversity Conservation and Ecological Security in the Yangtze River Basin Wuhu China

**Keywords:** Morphology, mutual genes, phylogenetics, taxonomy

## Abstract

Two new genera and seven new species of the subtribe Plexippina Simon, 1901 are reported from Southwest China based on both morphological and molecular evidence. They are *Chuanattus deelemanae* C. Wang, Mi & Li, **gen. et sp. nov.** (♂♀), *Dianattus proszynskii* C. Wang, Mi & Li, **gen. et sp. nov.** (♂♀), *Yaginumaella daolangi* C. Wang, Mi & Li, **sp. nov.** (♂♀), *Y. medog* C. Wang, Mi & Li, **sp. nov.** (♂♀), *Y. qianlei* C. Wang, Mi & Li, **sp. nov.** (♂♀), *Y. spinapophysis* C. Wang, Mi & Li, **sp. nov.** (♂♀), and *Y. xiaoqingi* C. Wang, Mi & Li, **sp. nov.** (♂♀). Diagnostic photos and a distributional map of those new species are provided. The genus *Yaginumaella* Prószyński, 1979 is reviewed, and 21 new and 34 restored combinations transferred from *Ptocasius* Simon, 1885 except *Y. pentamaculata* (Hu, 2001), **comb. nov.** from *Menemerus* Simon, 1868, are proposed: *Thyene incognita* (Żabka, 1981), **comb. nov.**, *Y. angulata* (Yang & Peng, 2023), **comb. nov.**, *Y. badongensis* Song & Chai, 1992, **comb. rest.**, *Y. bhutanica* Żabka, 1981, **comb. rest.**, *Y. bulbosa* Peng, Tang & Li, 2008, **comb. rest.**, *Y. cambridgei* Żabka, 1981, **comb. rest.**, *Y. circula* (Yang & Peng, 2023), **comb. nov.**, *Y. danzhu* (Yang & Peng, 2023), **comb. nov.**, *Y. davidi* (Yang & Peng, 2023), **comb. nov.**, *Y. falcata* Zhu, Zhang, Zhang & Chen, 2005, **comb. rest.**, *Y. filiforma* (Yang & Peng, 2023), **comb. nov.**, *Y. foliolata* (Yang & Peng, 2023), **comb. nov.**, *Y. gemina* (Yang & Peng, 2023), **comb. nov.**, *Y. gogonaica* Żabka, 1981, **comb. rest.**, *Y. helvetorum* Żabka, 1981, **comb. rest.**, *Y. hubeiensis* Li, Wang, Irfan & Peng, 2018, **comb. rest.**, *Y. hybrida* Żabka, 1981, **comb. rest.**, *Y. intermedia* Żabka, 1981, **comb. rest.**, *Y. jietouensis* (Yang & Peng, 2023), **comb. nov.**, *Y. linzhiensis* (Hu, 2001), **comb. nov.** (placed initially in *Ptocasius*), *Y. longapophysis* (Yang & Peng, 2023), **comb. nov.**, *Y. longlingensis* (Yang & Peng, 2023), **comb. nov.**, *Y. lushiensis* Zhang & Zhu, 2007, **comb. rest.**, *Y. montana* Żabka, 1981, **comb. rest.**, *Y. nepalica* Żabka, 1980, **comb. rest.**, *Y. nobilis* Żabka, 1981, **comb. rest.**, *Y. nova* Żabka, 1981, **comb. rest.**, *Y. orientalis* Żabka, 1981, **comb. rest.**, *Y. originalis* Żabka, 1981, **comb. rest.**, *Y. pilosa* Żabka, 1981, **comb. rest.**, *Y. pseudoflexa* Liu, Yang & Peng, 2016, **comb. rest.**, *Y. pulchella* Li, Wang, Irfan & Peng, 2018, **comb. rest.**, *Y. rectangula* (Yang & Peng, 2023), **comb. nov.**, *Y. robusta* (Yang & Peng, 2023), **comb. nov.**, *Y. senchalensis* Prószyński, 1992, **comb. rest.**, *Y. silvatica* Żabka, 1981, **comb. rest.**, *Y. simoni* Żabka, 1981, **comb. rest.**, *Y. songi* (Logunov, 1995), **comb. nov.**, *Y. stemmleri* Żabka, 1981, **comb. rest.**, *Y. strandi* Żabka, 1981, **comb. rest.**, *Y. subhubeiensis* (Wang, Mi & Peng, 2023), **comb. nov.**, *Y. supina* Żabka, 1981, **comb. rest.**, *Y. tenella* Żabka, 1981, **comb. rest.**, *Y. tengchongensis* (Yang & Peng, 2023), **comb. nov.**, *Y. tenzingi* Żabka, 1980, **comb. rest.**, *Y. thakkholaica* Żabka, 1980, **comb. rest.**, *Y. thimphuica* Żabka, 1981, **comb. rest.**, *Y. umbellulata* (Yang & Peng, 2023), **comb. nov.**, *Y. urbanii* Żabka, 1981, **comb. rest.**, *Y. variegata* (Logunov, 1995), **comb. nov.**, *Y. versicolor* Żabka, 1981, **comb. rest.**, *Y. wangdica* Żabka, 1981, **comb. rest.**, *Y. wuermli* Żabka, 1981, **comb. rest.**, *Y. zonata* (Yang & Peng, 2023), **comb. nov.***Pancorius lobatus* (Peng, Tso & Li, 2002), **comb. nov.** is also proposed (transferred from *Yaginumaella* and it could be a junior synonym of *Pancorius submontanus* Prószyński, 1992). *Ptocasius zabkai* Yang & Peng, 2023 is assigned as a synonym of *Y. zonata*.

## ﻿Introduction

Plexippina Simon, 1901, one of the most species-rich subtribes of the family Salticidae, currently contains 593 species under 35 genera primarily distributed in the Old World (WSC 2025). The taxonomic study of this subtribe remains unsatisfactory because more than half of its species are known only from a single-sex or juveniles, nearly one-fifth of its species lack valid diagnostic drawings, making them unable to be acutely recognized, and the presence of several potential polyphyletic and poorly defined genera (Kanesharatnam and Benjamin 2021; Wang et al. 2023b, 2024a; Marathe et al. 2024; WSC 2025). To date, 147 species in 17 genera have been reported from China, whose species number far exceeds that of other species-rich countries, such as India (48 species in 19 genera) and Indonesia (48 species in 13 genera) (WSC 2025).

*Yaginumaella* Prószyński, 1979 (type species *Y. ususudi* (Yaginuma, 1972)) has always been considered to be related to *Ptocasius* Simon, 1895 (type species *P. weyersi* Simon, 1885) and even unofficially viewed as a synonym of the latter (Żabka 1985; Logunov and Jäger 2015). Patoleta et al. (2020) transferred 37 *Yaginumaella* species to *Ptocasius* based on the similarity of the copulatory organs. However, this decision did not consider other vital characters, such as the habitus pattern, and thus, it has always been controversial (Logunov 2024; Wang et al. 2023b, 2024a, 2024b). Subsequently, many species sharing identical morphological characters, no matter the habitus or copulatory organs, were described and assigned into those two genera without a unified criterion, further confusing their taxonomy and phylogenetics (WSC 2025).

In our recent examination of jumping spiders on Plexippina from Southwest China, seven species were recognized as new to science, including two assigned to two new genera. Detailed descriptions for those new taxa are provided herein. A phylogenetic analysis based on five genes was also conducted to reveal the phylogenetic relationship of *Ptocasius*, *Yaginumaella* and the new genera.

## ﻿Materials and methods

### ﻿Species sampling and preservation

Specimens were collected by fogging or beating shrubs. They were preserved in 95% ethanol. Specimens are deposited in the Institute of Zoology, Chinese Academy of Sciences in Beijing (**IZCAS**), China, and Tongren University (**TRU**) in Tongren, China.

### ﻿Molecular data

To assess the taxonomic position of the described species in this study within the Plexippina, 24 individuals from 14 species were picked out from the examined materials for molecular sequencing. Their legs were used to extract genomic DNA and sequence five gene fragments: 16S rRNA–tRNA^Leu(CUN)^–NAD1, 18S, 28S, COI, and H3. Primer pairs are given in Table 1. Whole genomic DNA was extracted from tissue samples with the TIANamp Genomic DNA Kit (TIANGEN) following the manufacturer′s protocol for animal tissue. PCR amplification included a 2-min 95 °C initial denaturation and 35 iterations of 30 s at 95 °C, 30 s annealing steps at 46 °C (16S rRNA–tRNA^Leu(CUN)^–NAD1), 48 °C (CO1), 50 °C (18S), 56 °C (H3), and 62 °C (28S), 30 s at 72 °C, and one 10-min extension step at 72 °C. Sequencing of the PCR products was performed at Tsingke (Changsha, China).

**Table 1.** Primers and amplification conditions used in PCR.

Locus	Annealing temperature/time	Direction	Prime	Sequence5′–3′	Reference
16S rRNA–tRNA^Leu(CUN)^–NAD1	46°/30s	F	N1-J-12261	TCRTAAGAAATTATTTGAGC	Hedin 1997
		R	Faw16s2	GCACCTCGATGTTGGATTAA	Simon et al. 1994
18S	50°/30s	F	1F	TACCTGGTTGATCCTGCCAGTAG	Giribet and Ribera 2000
		R	5R	CTTGGCAAATGCTTTCGC	Giribet et al. 1996
28S	62°/30s	F	28SO	GAAACTGCTCAAAGGTAAACGG	Whiting et al. 1997
		R	28SC	GGTTCGATTAGTCTTTCGCC	
CO1	48°/30s	F	CO1 1628	ATAATGTAATTGTTACTGCTCA	Simon et al. 1994
		R	C1-N-2191	CCCGGTAAAATTAAAATATAAA	Dallas et al. 2005
H3	56°/30s	F	H3aF	ATGGCTCGTACCAAGCAGACV	Colgan et al. 1998
		R	H3aR	ATATCCTTRGGCATRATRGTG	

A total of 47 individuals belonging to 36 salticid species were used for phylogenetic analysis (Table 2). The ingroup includes 27 known or undescribed and seven new Plexippina species. *Bianor maculatus* Thorell, 1890 and *Habronattus hirsutus* (Peckham & Peckham, 1888) were used as outgroups. Raw sequences were edited using Geneious R7 (https://www.geneious.com). Edited sequences were queried in the NCBI BLAST database.

**Table 2.** The salticid species and their DNA data for molecular phylogenetic analysis.

Species	Molecular voucher	28S	18S	H3	COI	16S	Source
*Anarrhotus fossulatus*	AS19.1319	PQ278946	PQ278921	PQ273890	PQ305882		NCBI
*Bianor maculatus*	NZ19.9864	PQ278944	PQ278931	PQ273888	PQ305892		NCBI
*Colopsus cancellatus*	IFS_SAL_360	MN888680.1	MN888692.1	MN895432.1	MN895417.1		NCBI
*Colopsus ferruginus*	IFS_SAL_233	MN888672.1	MN888689.1	MN895429.1	MN895411.1		NCBI
*Colopsus magnus*	IFS_SAL_832	MN888671.1	MN888687.1		MN895408.1		NCBI
*Evarcha bulbosa*	AS19.2123		PQ278927	PQ273896	PQ305888		NCBI
*Evarcha albaria*		JN817038.1	JN816834.1		JN817256.1		NCBI
*Evarcha coreana*		JN817039.1	JN816835.1		JN817257.1		NCBI
*Evarcha latus*		MN888669		MN895427	MN895406		NCBI
*Ghatippus paschima*	IBC-BP833	PQ278962	PQ278937	PQ273906	PQ305897		NCBI
*Habronattus hirsutus*	IDWM.21018	PQ278948	PQ278923	PQ273892	PQ305884		NCBI
*Hyllus keratodes*	DDKM21.028	PQ278942	PQ278917	PQ273887	PQ305879		NCBI
*Pancorius alboclypeus*	IFS_SAL_1145	MN888667	MN888685	MN895424	MN895404		NCBI
*Pancorius altus*	IFS_SAL_1074	MN888666		MN895422	MN895403		NCBI
*Pancorius athukoralai*	IFS_SAL_1048	MN888663	MN888683	MN895421	MN895401		NCBI
*Pancorius dentichelis*	SWK12-0042	PQ278957	PQ278932	PQ273901	PQ305893		NCBI
*Pancorius petoti*	SWK12-0195	PQ278939	PQ278914	PQ273884	PQ305876		NCBI
*Plexippus paykulli*	AS19.7337	PQ278941	PQ278916	PQ273886	PQ305878		NCBI
*Ptocasius weyersi*	DDKM21.069	PQ278961	PQ278936	PQ273905			NCBI
*Telamonia festiva*	DDKM21.048	PQ278954	PQ278929	PQ273898	PQ305890		NCBI
*Tenkana arkavathi*	IBC-BX509	PQ278950	PQ278925	PQ273894	PQ305886		NCBI
*Tenkana manu*	IBC-BX510	PQ278956	PQ278919	PQ273900	PQ305880		NCBI
*Yaginumaella medvedevi*		JN817060.1	JN816858.1		JN817280.1		NCBI
*Anarrhotus fossulatus*	MS-397	PX367276	PX367259		PX367074		present study
*Chuanattus deelemanae*	MS-385	PX367272			PX367069	PX376930	present study
*Chuanattus deelemanae*	MS-386	PX367273	PX367255		PX367070	PX376931	present study
*Dexippus kleini*	MS-392		PX367257		PX367072		present study
*Dianattus proszynskii*	MS-347		PX367250	PX376941	PX367063	PX376927	present study
*Dianattus proszynskii*	MS-348		PX367251		PX367064	PX376928	present study
*Dianattus proszynskii*	MS-393	PX367275	PX367258	PX376948	PX393525	PX376933	present study
Evarcha cf. albaria	MS-403	PX367277	PX367260	PX376949	PX367077		present study
*Hyllus qishuoi*	MS-240		PX367246			PX376924	present study
*Hyllus qishuoi*	MS-315	PX367264	PX367248			PX376925	present study
*Nigorella mengla*	MS-346	PX367265	PX367249	PX376940	PX367062	PX376926	present study
*Ptocasius paraweyersi*	MS-391	PX367274	PX367256	PX376947	PX367071	PX376932	present study
*Ptocasius paraweyersi*	MS-394				PX367073	PX376934	present study
*Ptocasius strupifer*	MS-311	PX367263	PX367247	PX376939	PX367061		present study
*Yaginumaella daolangi*	MS-381	PX367268	PX367253	PX376944	PX393524		present study
*Yaginumaella medog*	MS-407	PX367278	PX367261	PX376950	PX367078	PX376937	present study
*Yaginumaella medog*	MS-408	PX367279	PX367262		PX367079	PX376938	present study
*Yaginumaella qianlei*	MS-401				PX367075	PX376935	present study
*Yaginumaella qianlei*	MS-402				PX367076	PX376936	present study
*Yaginumaella spinapophysis*	MS-382	PX367269		PX376945	PX367066		present study
*Yaginumaella spinapophysis*	MS-383	PX367270		PX376946	PX367067		present study
*Yaginumaella spinapophysis*	MS-384	PX367271	PX367254		PX367068		present study
*Yaginumaella xiaoqingi*	MS-379	PX367266		PX376942	PX367065		present study
*Yaginumaella xiaoqingi*	MS-380	PX367267	PX367252	PX376943			present study

Sequences were aligned by MAFFT (Katoh and Standley 2013) and were concatenated by the Concatenate in Phylosuite (Zhang et al. 2020). ModelFinder (Kalyaanamoorthy et al. 2017) was used to identify the best-fit models of molecular evolution for each locus: CO1: GTR+F+I+G4; 16S rRNA–tRNA^Leu(CUN)^–NAD1: TIM2+F+G4; 18S: K2P+I; 28S, and H3: GTR+F+R2.

The maximum likelihood (ML) tree was constructed using IQ-TREE 2.0 (Minh et al. 2020) with TBR (Tree-Bisection-Reconnection) branch swapping and 10000 bootstrap replicates with default parameters, and was final edited and dealt with in the iTOL (https://itol.embl.de/).

### ﻿Morphological data

Methods of specimen examination and photo and distributional map generation followed Wang et al. (2024a). All measurements are given in millimeters. Leg measurements are given as total length (femur, patella, tibia, metatarsus, tarsus). References to figures in the cited papers are listed in lowercase type (fig. or figs), and figures in this paper are noted with an initial capital (Fig. or Figs). Abbreviations used in the text and figures are as follows: **AERW** anterior eye row width; **ALE** anterior lateral eye; **AME** anterior median eye; **At** atrium; **CD** copulatory duct; **CO** copulatory opening; **E** embolus; **ED** embolic division; **EFL** eye field length; **FD** fertilization duct; **H** epigynal hood; **PERW** posterior eye row width; **PL** posterior tegular lobe; **PME** posterior median eye; **PLE** posterior lateral eye; **RTA** retrolateral tibial apophysis; **S** spermatheca; **SD** sperm duct; **SS**/**AG** secondary spermatheca/accessory gland; **TP** tegular process.

## ﻿Results

### ﻿Phylogenetic analysis

**Figure 1.**
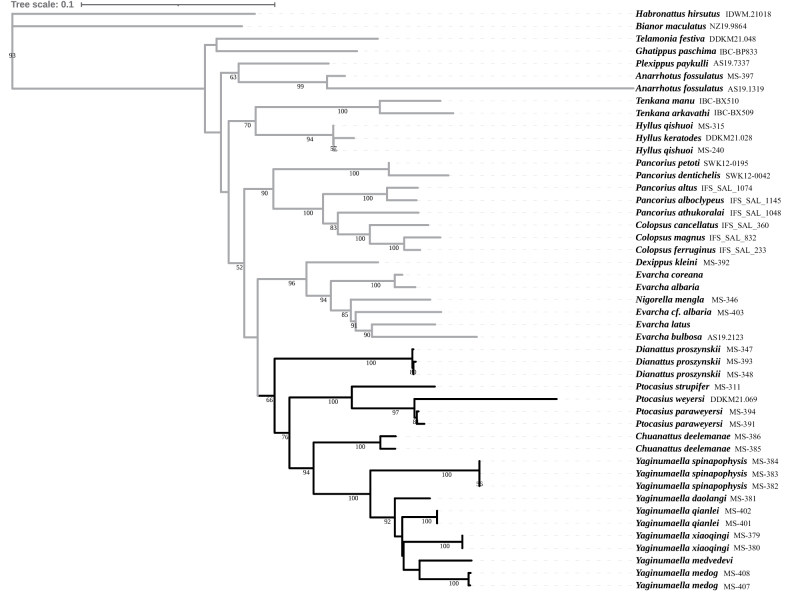
Maximum-likelihood tree based on five genes. The numbers at the nodes represent bootstrap values and are not displayed when they are less than 50. The species related to the present study are marked with dark color, and others with grey.

The concatenation of aligned sequences of the five genes resulted in a 2649 bp matrix. The total length of individual gene matrices was as follows: CO1 (505 bp, 43 individuals), 28s (690 bp, 39 individuals), 18s (793 bp, 38 individuals), H3 (253 bp, 31 individuals) and 16S (408 bp, 14 individuals). The ML phylogenetic analysis of the five-gene combined dataset retrieved a single tree. The phylogenetic results are shown in Fig. 1 and are generally consistent with Kanesharatnam and Benjamin (2021) and Marathe et al. (2024). In the tree, *Chuanattus* gen. nov. (represented by one species), *Dianattus* gen. nov. (represented by one species), *Ptocasius* Simon, 1885 (represented by three species), and *Yaginumaella* Prószyński, 1979 (represented by six species) form a single clade. The relationship of the genera in this clade could be uncertain, as reflected in the low bootstrap values of some nodes. However, the represented species for each genus are nested in separate branches, respectively, supporting that they should belong to different genera, and the result is consistent with our morphological identification. The type species of *Yaginumaella* has not been included because of a lack of specimens, and no sequences can be downloaded. However, among the six selected species, *Y. daolangi* sp. nov., *Y. qianlei* sp. nov., and *Y. xiaoqingi* sp. nov. share identical habitus and very similar copulatory organs to the type species, which well supports that they are congeners to the latter. Based on the above, we describe two new genera and propose the restored and new combinations.

### ﻿Taxonomy

#### ﻿Family Salticidae Blackwall, 1841

##### ﻿Genus Chuanattus

Taxon classificationAnimaliaAraneaeSalticidae

C. Wang, Mi & Li gen. nov.

DB80EA37-78BB-5A1D-A38E-CD2C13BE8BA8

https://zoobank.org/E86ED226-ADCB-48A5-AF35-E7F5D203E4A4

###### Type species.

*Chuanattus deelemanae* sp. nov.

###### Diagnosis.

**Figure 2.**
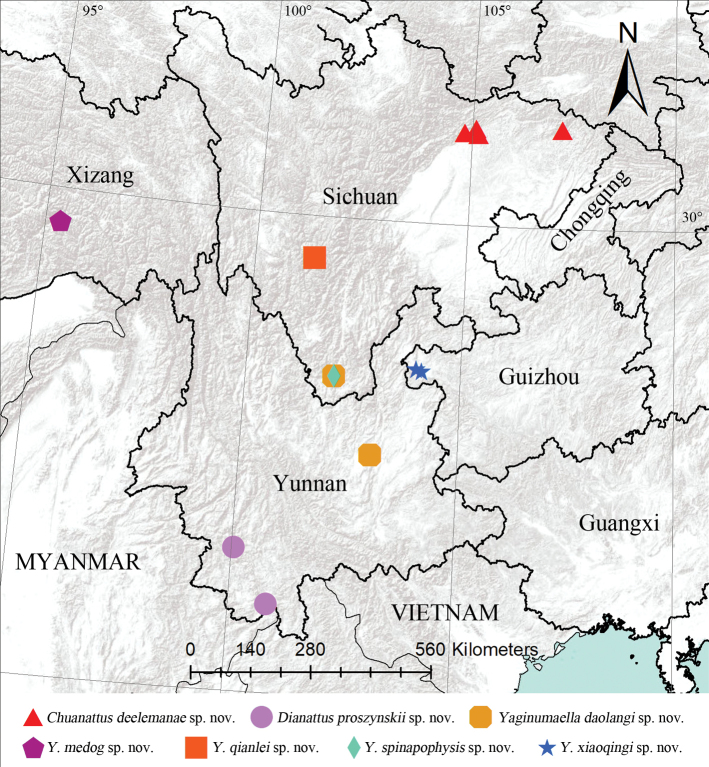
Geographical records of the new species.

**Figure 3.**
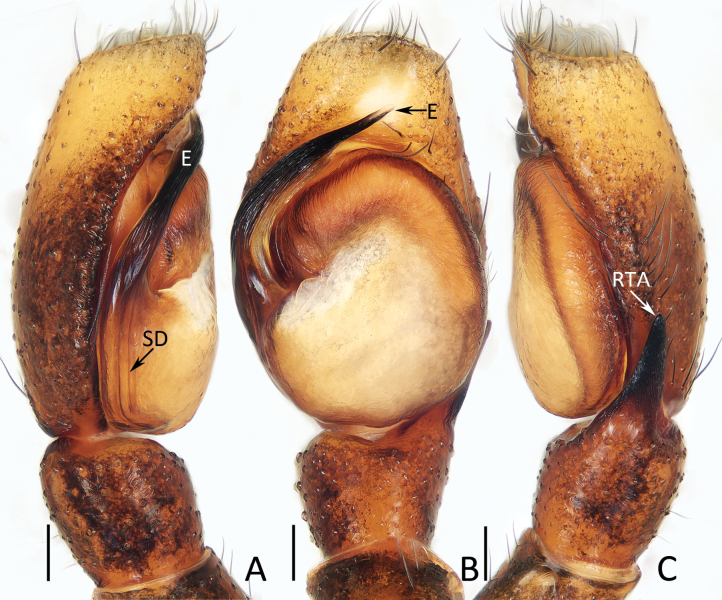
Male palp of *Chuanattus deelemanae* gen. et sp. nov., holotype (IZCAS-Ar45820). A. Palp, prolateral; B. Ditto, ventral; C. Ditto, retrolateral. Abbreviations: E = embolus, RTA = retrolateral tibial apophysis, SD = sperm duct. Scale bars: 0.1 mm.

**Figure 4.**
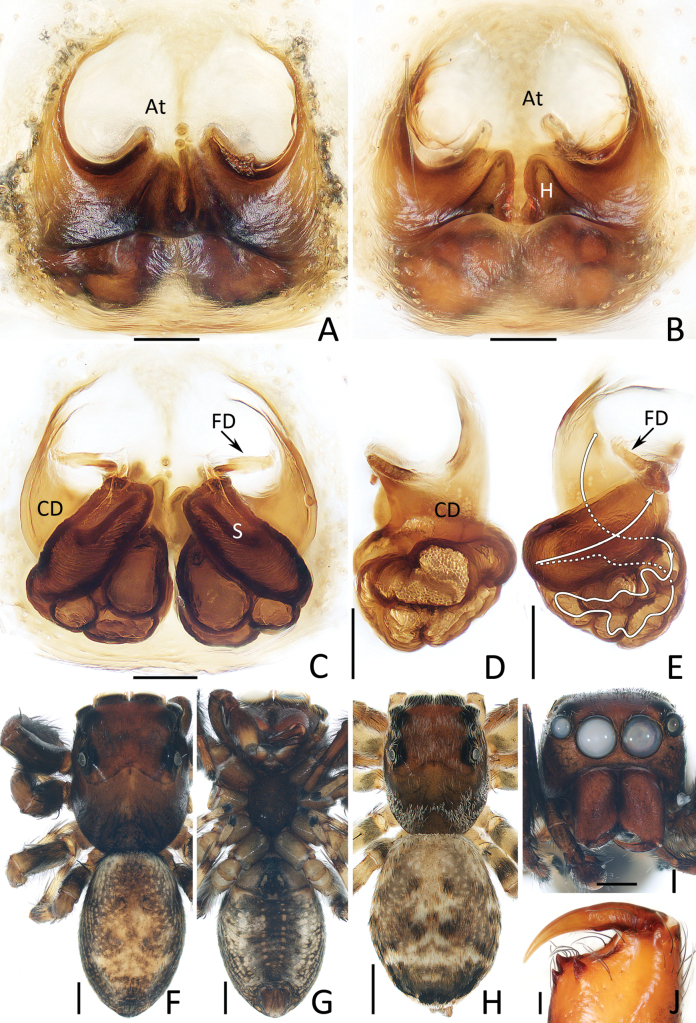
*Chuanattus deelemanae* gen. et sp. nov., male holotype (IZCAS-Ar45820) (F, G, I, J), female paratype (IZCAS-Ar45828) (A, C, H), and female paratype (IZCAS-Ar45829) (B, D, E). A, B. Epigyne, ventral; C. Vulva, dorsal; D. Copulatory duct, ventral; E. Copulatory duct, spermatheca and fertilization duct, dorsal; F, H. Habitus, dorsal; G. Ditto, ventral; I. Carapace, frontal; J. Chelicera, posterior. Abbreviations: At = atrium, CD = copulatory duct, FD = fertilization duct, H = epigynal hood, S = spermatheca. Scale bars: 0.1 mm (A–E, J); 0.5 mm (F–I).

*Chuanattus* gen. nov. resembles that of *Yaginumaella* Prószyński, 1979 in having a similar habitus and almost identical copulatory organs, especially the presence of a longitudinal, irregular central patch dorsally on the abdomen, a pair of epigynal hoods and similar path of copulatory ducts, but differs in: 1) the lacking of the posterior tegular lobe (Fig. 3B) vs present in *Yaginumaella* (Figs 7B, 9B, 11B, 13B, 15C); 2) the lacking of a longitudinal, central thoracal stripe (Fig. 4F, H) vs present except some males in *Yaginumaella* (Figs 8C, E, 10E, F, 12C, D, 16C, E); 3) the presence of a dorsal abdominal scutum in male (Fig. 4F) vs absent in *Yaginumaella* (Figs 8C, 10E, 12C, 14E, 16C). The genus also somewhat resembles that of *Ptocasius* Simon, 1895 in the general shape of copulatory organs, but can be distinguished by the followings: 1) the embolus is curved approximately a quarter of a circle (Fig. 3B) vs curved at least half a circle in *Ptocasius* (Żabka 1985: figs 513, 517, 521, 530; Logunov and Jäger 2015: fig. 52; Cao et al. 2016: fig. 38C, D; Patoleta et al. 2020: fig. 7G, H); 2) the lacking of the tegular bump (Fig. 3B), vs present except modified into a pale area in the type species and its congeners in *Ptocasius* (Żabka 1985: figs 513, 517, 521; Cao et al. 2016: fig. 38C, D; Patoleta et al. 2020: fig. 7G, H); 3) the copulatory ducts are not forming median ridges (Fig. 2C, E) vs forming obvious prominent median ridges in *Ptocasius* (Żabka 1985: fig. 526; Logunov and Jäger 2015: fig. 55; Cao et al. 2016: fig. 39B; Patoleta et al. 2020: figs 8G, H, 9G, H); 4) the abdomen has a longitudinal, central irregular path dorsally (Fig. 4F, H) vs has an anterior, transverse, near arc-shaped stripe and a pair of medio-lateral or posterolateral, oval patches or instead by a complete transverse patch in *Ptocasius* (Patoleta et al. 2020: figs 7A, 8A, 9A; Logunov 2024: figs 125, 130).

###### Description.

See description of type species.

###### Composition.

Currently, it only includes the type species.

###### Distribution.

China (Sichuan).

###### Etymology.

The generic name is a combination of chuan, the pinyin of a short Chinese name of Sichuan Province, the type locality of the type species, and attus, meaning jumper. The gender is masculine.

###### Comments.

*Yaginumaella pentamaculata* (Hu, 2001), comb. nov. shares a similar epigyne with the type species, especially the form of the atrium and the epigynal hood position, indicating it could be a potential member of this genus, but those are not obviously different from some *Yaginumaella*. Thus, it has been transferred to *Yaginumaella* temporarily because it is generally more similar to *Y. circula* (Yang & Peng, 2023), comb. nov.

##### ﻿ Chuanattus deelemanae

Taxon classificationAnimaliaAraneaeSalticidae

C. Wang, Mi & Li sp. nov.

1BD7F6BA-858E-565C-9D9D-5C3F4AB3D341

https://zoobank.org/834ECA29-4663-4DCE-84DE-3214BF2AB35F

Figs 3, 4

###### Type material.

***Holotype*** • ♂ (IZCAS-Ar45820), China: Sichuan: Jiangyou County, Yongsheng Township, Xinbei Village (31°56.25'N, 104°48.72'E, ca 680 m), 16.vi.2024, X.Q. Zhang, Y. Wang and Q.Z. Meng leg. ***Paratypes*** • 7♂15♀ (IZCAS-Ar45821–45842), same data as for holotype; • 6♂2♀ (IZCAS-Ar45843–45850), Jiangyou County, Erlangmiao Township, Beishang Village (32°1.77'N, 105°5.80'E, ca 640 m), 13.v.2024, same collectors as for holotype; • 17♂14♀ (IZCAS-Ar45851–45881), Zitong County, Hekou Township, junction of Hejiawan, Daozuomiao, and Dasheng Villages (31°54.65'N, 105°9.37'E, ca 750 m) 13.vi.2024, same collectors as for holotype; • 1♂1♀ (IZCAS-Ar45882–45883), Tongjiang County, Xinglong Township, Sanjiao Village (32°4.52'N, 107°13.44'E, ca 910 m), 16.vi.2024, same collectors as for holotype.

###### Diagnosis.

The male of *Chuanattus deelemanae* sp. nov. resembles that of *Yaginumaella striatipes* (Grube, 1861) in having a similar palp, especially the origination and shape of the embolus, but can be easily distinguished by the lack of the posterior lobe, and by the flat tegulum (Fig. 3B, C) vs the presence of a posterior lobe and the tegulum is swollen medio-posteriorly in *Y. striatipes* (Prószyński 1971: figs 28, 29). The female of this new species resembles that of *Y. pentamaculata* (Hu, 2001), comb. nov. in having a similar atrium, centrally located epigynal hoods close to each other, but can be easily distinguished by the maximum distance between epigynal hoods, which is > 3/4 the epigynal width (Fig. 4A, B) vs just ~1/5 in *Y. pentamaculata* (Hu 2001: fig. 8-250-2).

###### Description.

**Male** (Figs 3A–C, 4F, G, I, J). Total length 5.03. Cephalothorax 2.53 long, 1.88 wide. Abdomen 2.62 long, 1.70 wide. Eye sizes and inter distances: AME 0.56, ALE 0.31, PLE 0.28, AERW 1.70, PERW 1.67, EFL 1.05. Legs: I 5.13 (1.45, 0.90, 1.30, 0.90, 0.58), II 4.41 (1.38, 0.80, 1.00, 0.73, 0.50), III 4.91 (1.53, 0.75, 1.08, 1.00, 0.55), IV 5.14 (1.55, 0.73, 1.13, 1.18, 0.55). Carapace dark red to dark, covered with dense dark and golden setae of various lengths; fovea dark, linear. Chelicerae dark red, with typical dentition (two promarginal and one retromarginal teeth). Endites broadened distally, with pale inner-distal portions. Labium dark. Sternum dark, spotted, with truncated anterior edge. Legs spiny, covered with dense setae ventrally on patellae and tibiae I. Dorsum of abdomen mainly dark brown, with central, big scutum and irregular, longitudinal, medio-posterior pale patch; venter mainly dark, with pair of central, dotted lines.

Palp (Fig. 3A–C): tibia almost as long as wide in retrolateral view, with strongly sclerotized, straight retrolateral apophysis slightly shorter than tibial length, and tapered to rather blunt tip; cymbium ~ 1.5× longer than wide, with truncated anterior edge; tegulum flat, almost round; embolus originates from ca 9 o′clock position, curved clockwise 1/4 circle and with blunt tip.

**Female** (Fig. 4A–E, H). Total length 6.20. Cephalothorax 2.71 long, 2.16 wide. Abdomen 3.53 long, 2.67 wide. Eye sizes and inter distances: AME 0.59, ALE 0.33, PLE 0.31, AERW 1.84, PERW 1.84, EFL 1.18. Legs: I 4.88 (1.50, 0.95, 1.13, 0.75, 0.55), II 4.58 (1.40, 0.90, 1.03, 0.70, 0.55), III 5.46 (1.75, 0.95, 1.08, 1.05, 0.63), IV 5.79 (1.78, 0.88, 1.25, 1.25, 0.63). Habitus (Fig. 4H) similar to that of male except paler, carapace covered with dense pale setae and abdomen lacking dorsal scutum.

Epigyne (Fig. 4A–E): slightly wider than long, with pair of central epigynal hoods close to or touching each other; atrium anteriorly located, double oval; copulatory openings beneath lateral sides of atrium, with complicated coils; spermathecae elongated, anterior-oblique extending; fertilization ducts appear from anterior edges of spermathecae.

###### Distribution.

Known only from the type locality in Sichuan, China (Fig. 2).

###### Etymology.

The specific name is a patronym in honor of the late Dr Christa L. Deeleman-Reinhold, who contributed to the taxonomic study of spiders in Southeast Asia; noun (name) in genitive case.

##### ﻿Genus Dianattus

Taxon classificationAnimaliaAraneaeSalticidae

C. Wang, Mi & Li gen. nov.

5B4DB8F5-0806-54F0-84F9-81D5495CF3D9

https://zoobank.org/70227C90-C6DF-4C53-B318-9555DF3FD8C1

###### Type species.

*Dianattus proszynskii* sp. nov.

###### Diagnosis.

**Figure 5.**
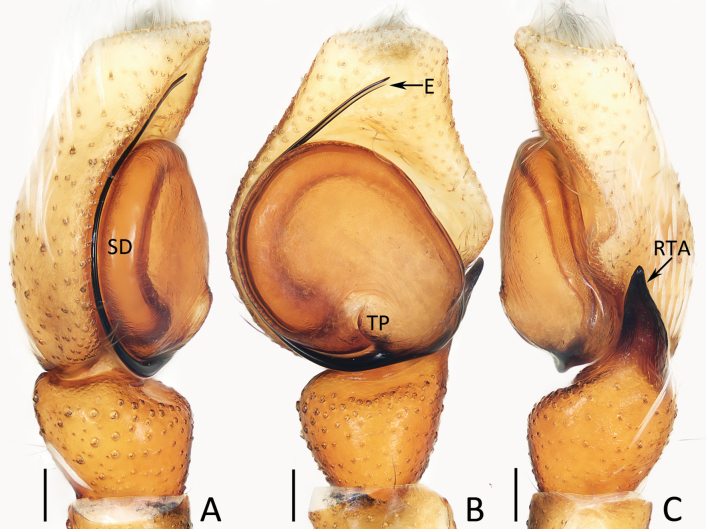
Male palp of *Dianattus proszynskii* gen. et sp. nov., holotype (TRU-JS 0831). A. Palp, prolateral; B. Ditto, ventral; C. Ditto, retrolateral. Abbreviations: E = embolus, RTA = retrolateral tibial apophysis, SD = sperm duct, TP = tegular process. Scale bars: 0.1 mm.

**Figure 6.**
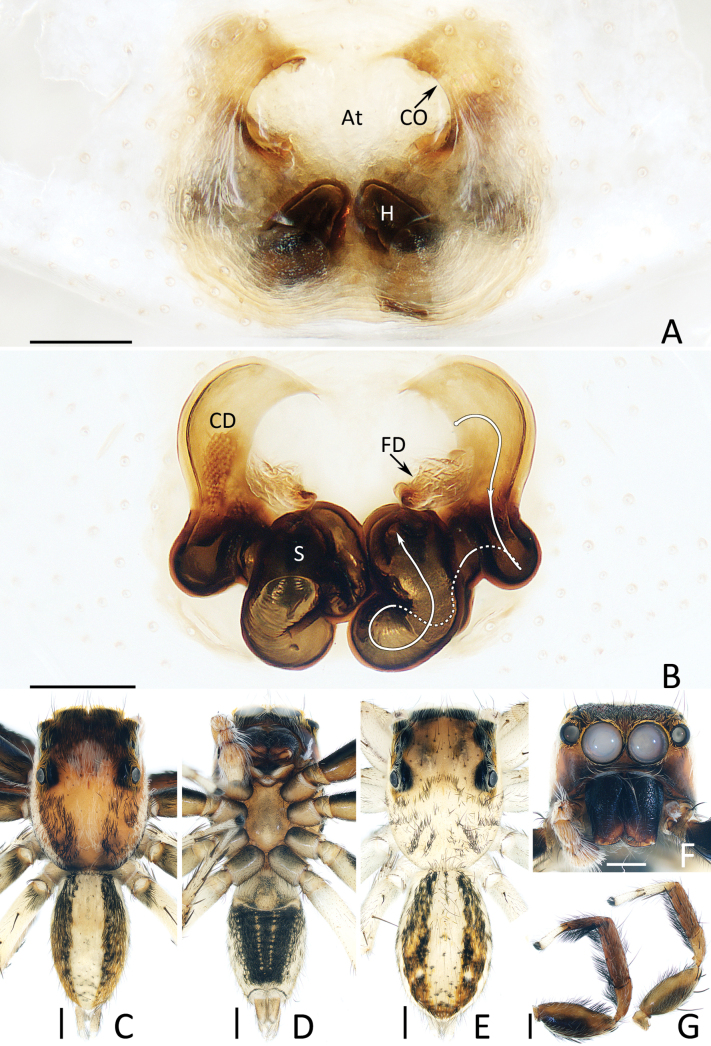
*Dianattus proszynskii* gen. et sp. nov., male holotype (TRU-JS 0831) (C, D, F, G) and female paratype (TRU-JS 0834) (A, B, E). A. Epigyne, ventral; B. Vulva, dorsal; C, E. Habitus, dorsal; D. Ditto, ventral; F. Carapace, frontal; G. Leg I, II, retrolateral. Abbreviations: At = atrium, CD = copulatory duct, CO = copulatory opening, FD = fertilization duct, H = epigynal hood, S = spermatheca. Scale bars: 0.1 mm (A, B); 0.5 mm (C–G).

**Figure 7.**
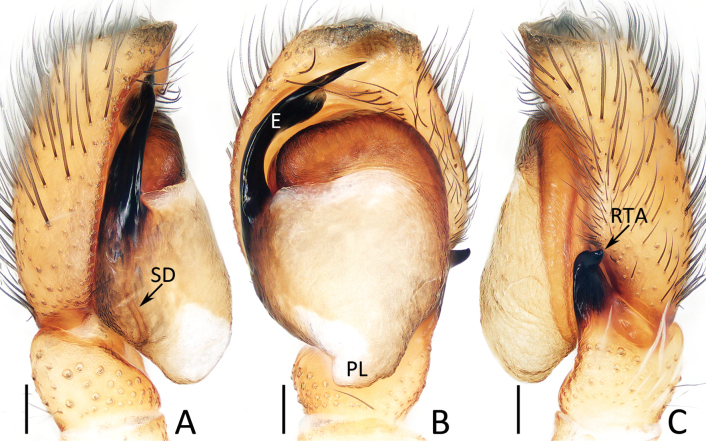
Male palp of *Yaginumaella daolangi* sp. nov., holotype (TRU-JS 0839). A. Palp, prolateral; B. Ditto, ventral; C. Ditto, retrolateral. Abbreviations: E = embolus, RTA = retrolateral tibial apophysis, PL = posterior tegular lobe, SD = sperm duct. Scale bars: 0.1 mm.

**Figure 8.**
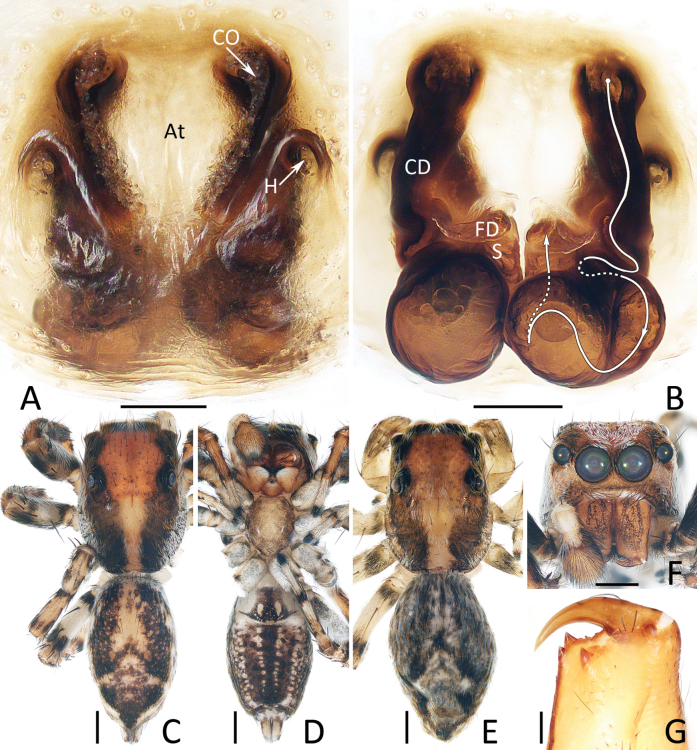
*Yaginumaella daolangi* sp. nov., male holotype (TRU-JS 0839) (C, D, F, G) and female paratype (TRU-JS 0840) (A, B, E). A. Epigyne, ventral; B. Vulva, dorsal; C, E. Habitus, dorsal; D. Ditto, ventral; F. Carapace, frontal; G. Chelicera, posterior. Abbreviations: At = atrium, CD = copulatory duct, CO = copulatory opening, FD = fertilization duct, H = epigynal hood, S = spermatheca. Scale bars: 0.1 mm (A, B, G); 0.5 mm (C–F).

**Figure 9.**
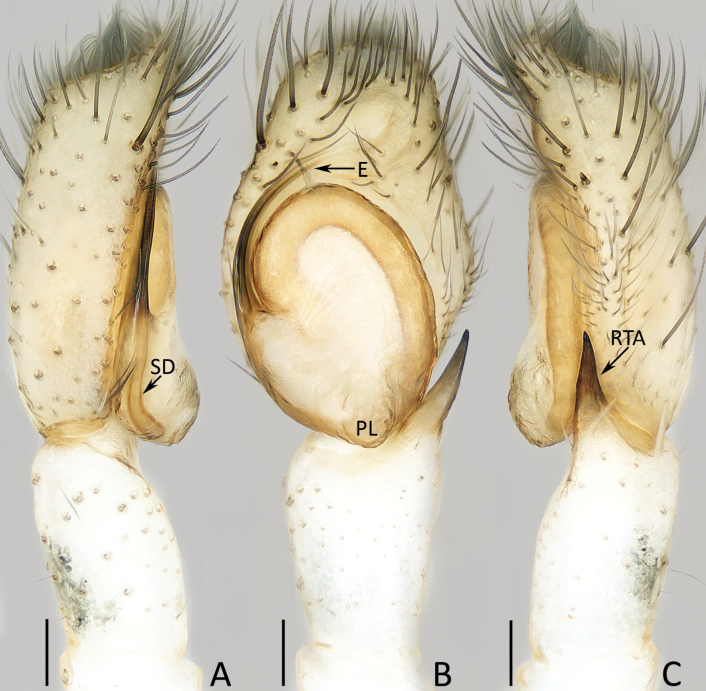
Male palp of *Yaginumaella medog* sp. nov., holotype (TRU-JS 0841). A. Palp, prolateral; B. Ditto, ventral; C. Ditto, retrolateral. Abbreviations: E = embolus, RTA = retrolateral tibial apophysis, PL = posterior tegular lobe, SD = sperm duct. Scale bars: 0.1 mm.

**Figure 10.**
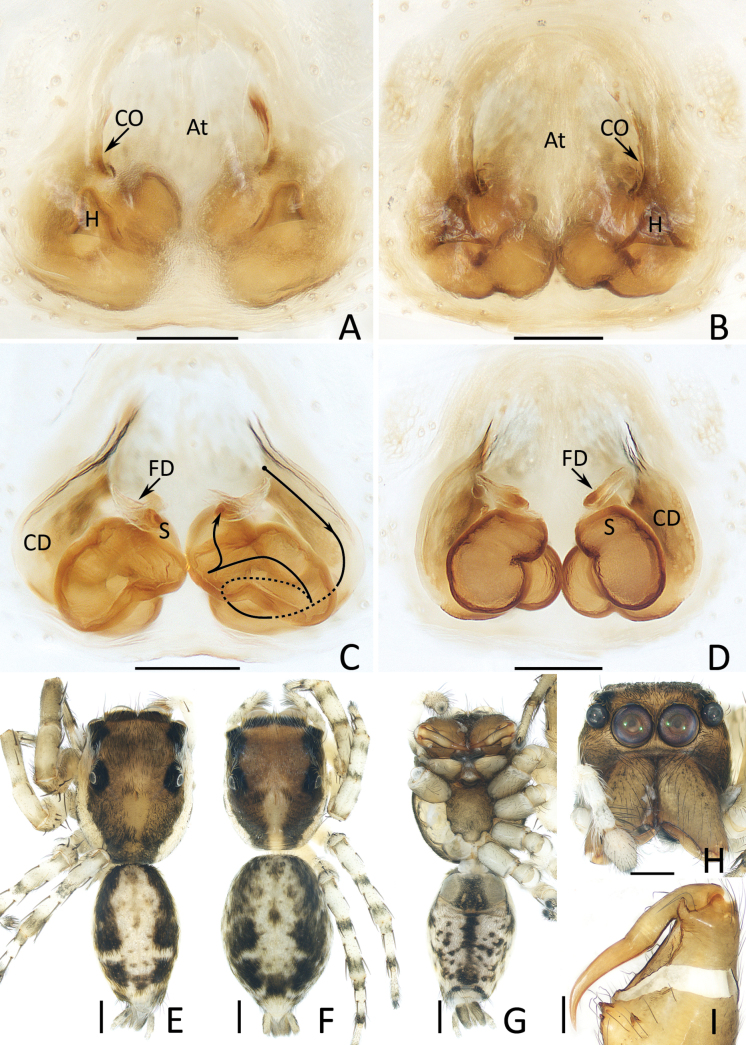
*Yaginumaella medog* sp. nov., male holotype (TRU-JS 0841) (E, G–I), female paratype (TRU-JS 0844) (A, C, F) and female paratype (TRU-JS 0845) (B, D). A, B. Epigyne, ventral; C, D. Vulva, dorsal; E, F. Habitus, dorsal; G. Ditto, ventral; H. Carapace, frontal; I. Chelicera, posterior. Abbreviations: At = atrium, CD = copulatory duct, CO = copulatory opening, FD = fertilization duct, H = epigynal hood, S = spermatheca. Scale bars: 0.1 mm (A–D); 0.2 mm (**I**); 0.5 mm (E–H).

**Figure 11.**
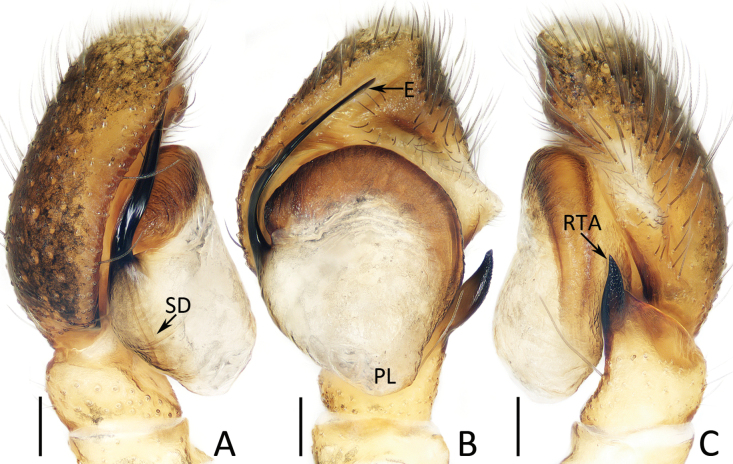
Male palp of *Yaginumaella qianlei* sp. nov., holotype (TRU-JS 0846). A. Palp, prolateral; B. Ditto, ventral; C. Ditto, retrolateral. Abbreviations: E = embolus, RTA = retrolateral tibial apophysis, PL = posterior tegular lobe, SD = sperm duct. Scale bars: 0.1 mm.

**Figure 12.**
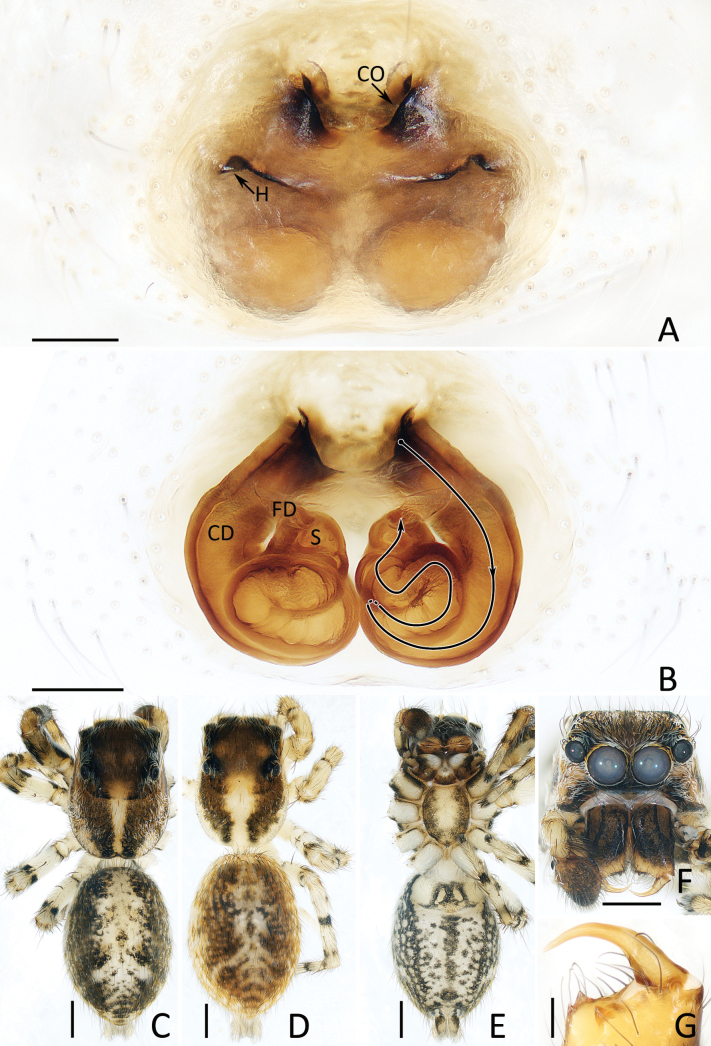
*Yaginumaella qianlei* sp. nov., male holotype (TRU-JS 0846) (C, E–G) and female paratype (TRU-JS 0848) (A, B, D). A. Epigyne, ventral; B. Vulva, dorsal; C, D. Habitus, dorsal; E. Ditto, ventral; F. Carapace, frontal G. Chelicera, posterior. Abbreviations: CD = copulatory duct, CO = copulatory opening, FD = fertilization duct, H = epigynal hood, S = spermatheca. Scale bars: 0.1 mm (A, B, G); 0.5 mm (C–F).

**Figure 13.**
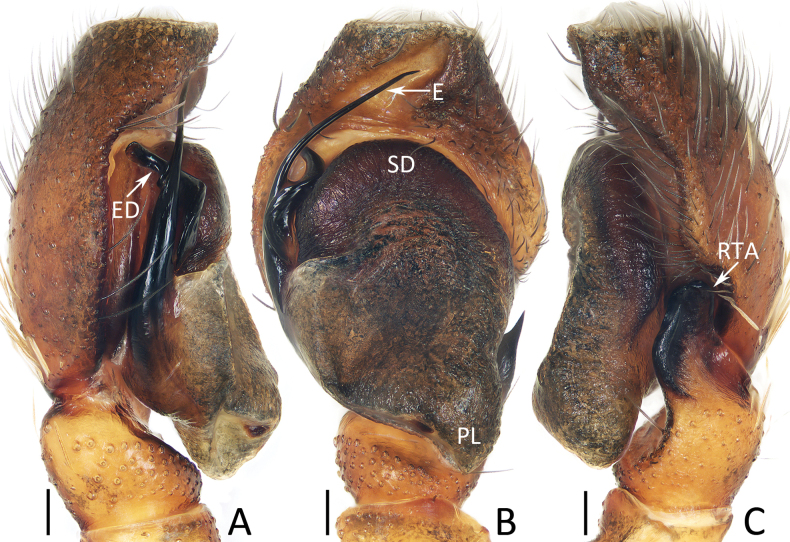
Male palp of *Yaginumaella spinapophysis* sp. nov., holotype (IZCAS-Ar45885). A. Palp, prolateral; B. Ditto, ventral; C. Ditto, retrolateral. Abbreviations: E = embolus, ED = embolic division, RTA = retrolateral tibial apophysis, PL = posterior tegular lobe, SD = sperm duct. Scale bars: 0.1 mm.

**Figure 14.**
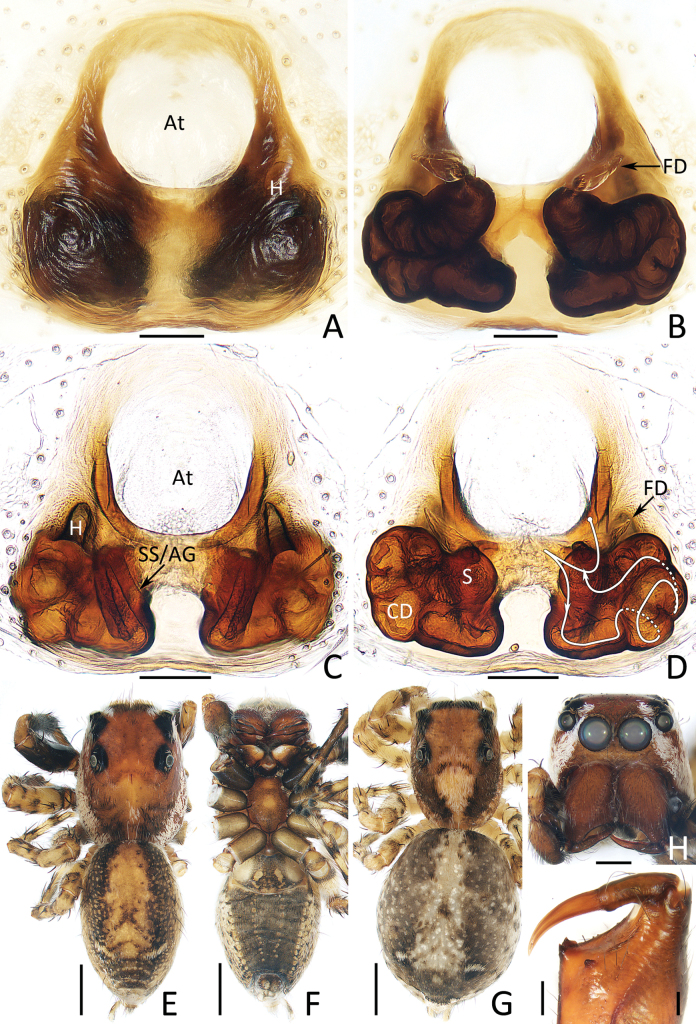
*Yaginumaella spinapophysis* sp. nov., male holotype (IZCAS-Ar45885) (E, F, H, I), female paratype (IZCAS-Ar45886) (A, B, G) and female paratype (IZCAS-Ar45887) (C, D). A, C. Epigyne, ventral; B, D. Vulva, dorsal; E, G. Habitus, dorsal; F. Ditto, ventral; H. Carapace, frontal; I. Chelicera, posterior. Abbreviations: At = atrium, CD = copulatory duct, FD = fertilization duct, H = epigynal hood, S = spermatheca, SS/AG = secondary spermatheca/accessory gland. Scale bars: 0.1 mm (A–D, I); 0.5 mm (H); 1.0 mm (E–G).

**Figure 15.**
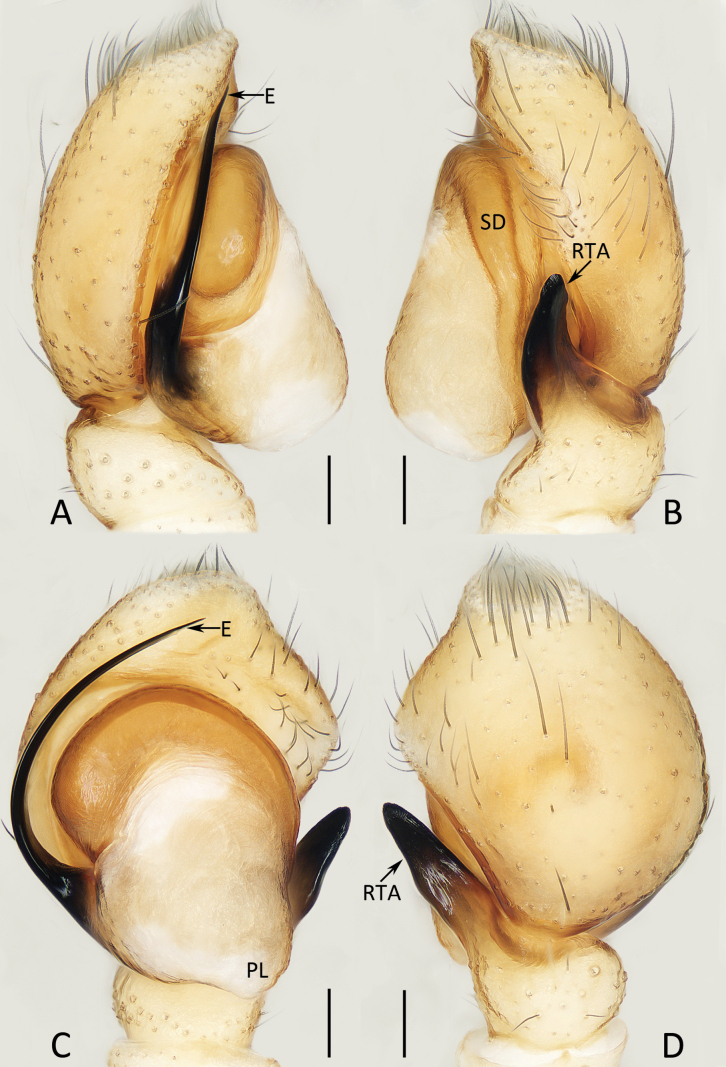
Male palp of *Yaginumaella xiaoqingi* sp. nov., holotype (IZCAS-Ar45889). A. Prolateral; B. Retrolateral; C. Ventral; D. Dorsal. Abbreviations: E = embolus, RTA = retrolateral tibial apophysis, PL = posterior tegular lobe, SD = sperm duct. Scale bars: 0.1 mm.

**Figure 16.**
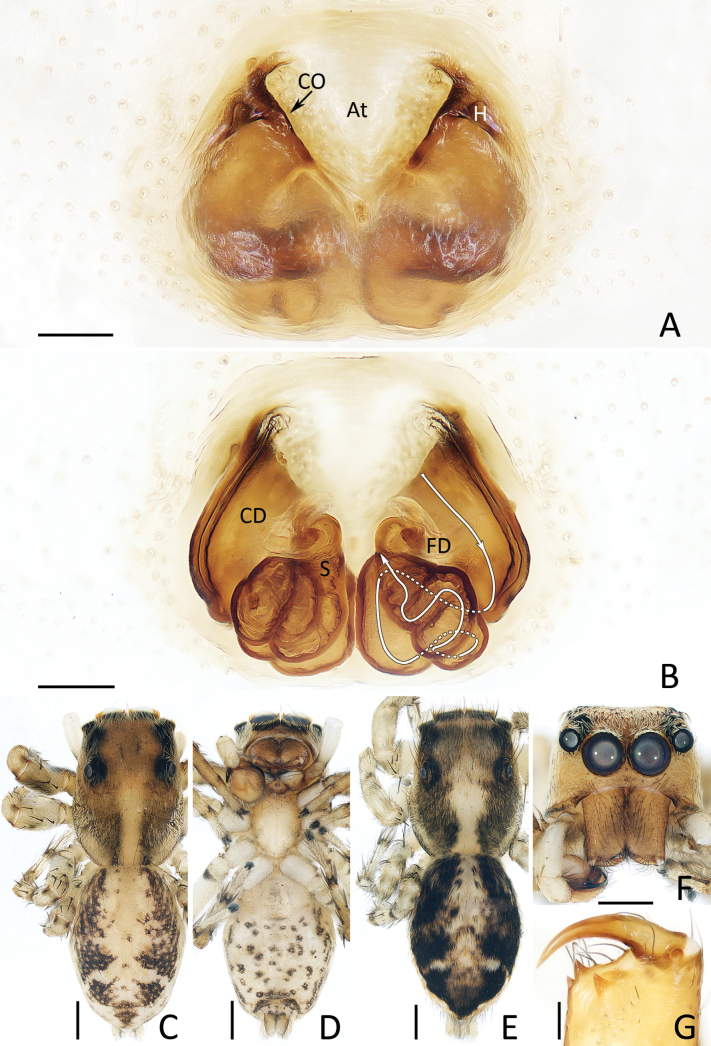
*Yaginumaella xiaoqingi* sp. nov., male holotype (IZCAS-Ar45889) (C, D, F, G) and female paratype (IZCAS-Ar45890) (A, B, E). A. Epigyne, ventral; B. Vulva, dorsal; C, E. Habitus, dorsal; D. Ditto, ventral; F. Carapace, frontal; G. Chelicera, posterior. Abbreviations: At = atrium, CD = copulatory duct, CO = copulatory opening, FD = fertilization duct, H = epigynal hood, S = spermatheca. Scale bars: 0.1 mm (A, B, G); 0.5 mm (C–F).

*Dianattus* gen. nov. resembles that of *Yaginumaella* Prószyński, 1979 in having a very similar habitus and epigynal structure, but differs in: 1) the tegulum has a process near the embolic base and lacking a posterior lobe (Fig. 5B) vs lacking similar process and having a posterior lobe in *Yaginumaella* (Figs 7B, 9B, 11B, 13B, 15C); 2) the presence of clusters of ventral dense setae on metatarsi I, femora I, II, patellae I, II, and tibiae I, II (Fig. 6G) vs absent in *Yaginumaella* (Żabka 1981: fig. 3; Wang et al. 2023a: 23A; Wang et al. 2024b: fig. 46C); 3) the copulatory ducts are forming arc-shaped portions on the bilateral sides of copulatory openings (Fig. 6B) vs posteriorly extending from the origin in *Yaginumaella* (Figs 8B, 10C, D, 12B, 14B, D, 16B).

###### Description.

See description of type species.

###### Composition.

The genus is monotypic presently.

###### Distribution.

China (Yunnan).

###### Etymology.

The generic name is a combination of dian, the pinyin of a short Chinese name of Yunnan Province, the type locality of the type species, and attus, meaning jumper. The gender is masculine.

###### Comments.

*Yaginumaella pilosa* Żabka, 1981 comb. rest. shares a very similar palpal structure with the type species, which indicates it could be a potential member of the genus. However, its generic position cannot be confirmed entirely due to the lack of other essential characteristics, such as habitus. Based on that, it has not been transferred into the genus, but further taxonomic attention is necessary.

##### ﻿ Dianattus proszynskii

Taxon classificationAnimaliaAraneaeSalticidae

C. Wang, Mi & Li sp. nov.

3048D0B9-3D72-5ACD-A59B-8FAD4E028EEA

https://zoobank.org/8D313903-BDCC-4D80-8F94-488FB14E0403

Figs 5, 6

###### Type material.

***Holotype*** • ♂ (TRU-JS 0831), China: Yunnan: Menghai County, Menghun Township (21°50.6'N, 100°51.87'E, ca 1,210 m), 27.xi.2024, H. Qiu leg. ***Paratypes*** • 2♂3♀ (TRU-JS 0832–0836), same data as for holotype; • 1♂1♀ (TRU-JS 0837–0838), Lancang Lahu Autonomous County, Donghe Township, Dadonghe Village (22°58.57'N, 100°4.2'E, ca 1,650 m), 18.iii.2024, H. Qiu leg.

###### Diagnosis.

The male of *Dianattus proszynskii* sp. nov. resembles that of *Yaginumaella pilosa* Żabka, 1981, comb. rest. in having a similar palpal structure, especially the origination of the embolus, flat tegulum, and the presence of a similar process near the embolic base, but can be easily distinguished by the apically blunt retrolateral tibial apophysis and truncated anterior cymbial edge in ventral view (Fig. 5B) vs pointed and not truncated in *Y. pilosa* (Żabka 1981: fig. 60). The female of the new species resembles that of *Y. rectangula* (Yang & Peng, 2023), comb. nov. in having a pair of epigynal hoods with similar locations and sizes, but can be easily distinguished by the path of copulatory ducts, which form arc-shaped portions on the lateral of the copulatory openings at the origin (Fig. 6B) vs posteriorly extending from the origin in *Y. rectangula* (Yang and Peng 2023: figs 27C, 28B).

###### Description.

**Male** (Figs 5A–C, 6C, D, F, G). Total length 4.78. Cephalothorax 2.50 long, 1.94 wide. Abdomen 2.25 long, 1.31 wide. Eye sizes and inter distances: AME 0.56, ALE 0.31, PLE 0.29, AERW 1.69, PERW 1.72, EFL 1.06. Legs: I 5.44 (1.63, 0.93, 1.45, 0.98, 0.45), II 4.69 (1.50, 0.83, 1.10, 0.83, 0.43), III 5.34 (1.70, 0.83, 1.08, 1.15, 0.58), IV 5.64 (1.68, 0.75, 1.30, 1.33, 0.58). Carapace mainly dark yellow, covered with sparse, long, dark setae and dense, much shorter orange, dark and pale setae, with pair of submarginal white setal bands; fovea longitudinal, red-brown. Chelicerae red-brown to dark, with typical dentition. Endites yellow, with pale inner portions. Labium darker than endites. Sternum yellow except central portion mingled with brown. Legs pale to red-brown, with clusters of ventral dense setae on metatarsi I, femora I, II, patellae I, II, and tibiae I, II. Dorsum of abdomen with longitudinal, central pale stripe extending across whole surface; venter dark, with pair of yellow dotted lines centrally.

Palp (Fig. 5A–C): tibia slightly wider than long; retrolateral tibial apophysis tapered, somewhat less than tibial length, extending upward to rather pointed tip slightly curved inward; cymbium ~1.25× longer than wide, with truncated anterior edge; tegulum almost oval, flat, with half-round process near embolic base; embolus originates from ca 5:30 o′clock position, curved ~1/2 circle, and with blunt tip.

**Female** (Fig. 6A, B, E). Total length 4.76. Cephalothorax 2.38 long, 1.82 wide. Abdomen 2.41 long, 1.52 wide. Eye sizes and inter distances: AME 0.54, ALE 0.30, PLE 0.28, AERW 1.64, PERW 1.70, EFL 1.07. Legs: I 4.33 (1.25, 0.80, 1.20, 0.63, 0.45), II 4.07 (1.20, 0.73, 1.13, 0.58, 0.43), III 4.58 (1.43, 0.75, 1.00, 0.90, 0.50), IV 5.06 (1.55, 0.73, 1.15, 1.13, 0.50). Habitus (Fig. 6E) similar to that of male except paler carapace without lateral, white setal bands and dorsum of abdomen with pair of posterior, round, white setal spots.

Epigyne (Fig. 6A, B): slightly wider than long, with pair of bell-shaped hoods opened oblique posteriorly and close to each other at anterior-most edges; atrium almost oval, anteriorly located; copulatory openings slit-shaped; copulatory ducts forming arc-shaped portions lateral to copulatory openings at origin, then curved and twisted into irregular path; spermathecae sub-spherical, touched each other; fertilization ducts almost transversely extending.

###### Distribution.

Known only from the type locality in Yunnan, China (Fig. 2).

###### Etymology.

The species name is a patronym in honor of Prof. Jerzy Prószyński, who has significantly contributed to the taxonomy of salticid spiders worldwide; noun (name) in genitive case.

##### ﻿Genus Yaginumaella

Taxon classificationAnimaliaAraneaeSalticidae

Prószyński, 1979

46C400DE-CE69-5C0A-84F2-4E7F2C3CFF5A

###### Type species.

*Pellenes ususudi* Yaginuma, 1972 by original designation.

###### Diagnosis.

*Yaginumaella* resembles that of *Ptocasius* in having similar copulatory organs, but can be distinguished by the followings: 1) the presence of a posterior tegular lobe and lack of a tegular bump (Figs 7B, 9B, 11B, 13B, 15C) vs lacking of a tegular lobe and having a tegular bump (modified into a pale area in the type species and its congeners) near the embolic base in *Ptocasius* (Żabka 1985: figs 513, 517, 521; Cao et al. 2016: fig. 38C, D; Patoleta et al. 2020: fig. 7G, H); 2) the presence of a longitudinal, thoraical stripe (Figs 8C, E, 10E, F, 12C, D, 16C, E) vs absent in *Ptocasius* (Patoleta et al. 2020: figs 7A, 8A, 9A; Logunov 2024: figs 125, 130); 3) the copulartory ducts are diverse in path (Fig. 8B, 10C, D, 12B, 14B, D, 16B) vs very consistent, curved into almost U-shapes until forming median ridges, and then run posteriorly and continue to form distal coils encircled or around the distinct, elongate-oval sermathecae in *Ptocasius* (Żabka 1985: fig. 526; Cao et al. 2016: fig. 38B; Patoleta et al. 2020: fig. 8G, H, 9G, H). The female of *Yaginumaella* also somewhat resembles some of the species of *Thyene* Simon, 1885 mainly reported from East and South Asia, such as *T. yuxi* Xie & Peng, 1995, in having a similar habitus and epigynal structure, but can be distinguished by the diverse path of copulatory ducts (Figs 8B, 10C, D, 12B, 14B, D, 16B) vs relatively united, curved into almost U-shapes until forming median ridges, and then run posteriorly and continue to create several distal loops (Wang et al. 2023b: figs 49B, 51B); 2) the single retromarginal cheliceral tooth (see the below description of the *Yaginumaella* spp.) vs a bifurcated tooth with two cusps in those of *Thyene* species (see the description in Wang et al. 2023b).

###### Description.

Small to medium-sized spiders. Carapace almost square, and setose, mostly with pair of sub-marginal pale setal bands, and longitudinal, central stripe extending across thoracic surface. Chelicerae with typical dentition (two promarginal teeth and one retromarginal tooth), except *Y. medog* with two retromarginal teeth. Endites broadened distally, with pale disto-inner areas and disto-inner marginal dense setae. Labium tapered, covered with disto-marginal setae. Sternum almost shield-shaped, mostly with straight anterior edge. Legs vary in color and spiny. Dorsum of abdomen with longitudinal, central irregular patch; venter mostly with dotted lines.

Male palp: tibia mostly almost as long as wide except *Y. medog* with much longer tibia; retrolateral tibial apophysis mainly tapered, bifurcated or not; cymbium setose, with truncated or cambered anterior edge, and retrolateral process (just present in *Y. qianlei*), tegulum swollen medio-posteriorly, with obvious posterior lobe; sperm duct runs along tegular submargin, and gradually thinner from retrolateral most portion; embolus originates from posterior or prolateral portion of tegulum, curved clockwise, and mostly with pointed tip.

Epigyne: with pair of epigynal hoods with different shapes and situations key to species identification; atrium mainly anteriorly located, with various sizes, copulatory openings slit-shaped, beneath lateral portions of atrium, copulatory ducts forming different paths, secondary spermatheca/accessory glands appear in limited species (such as *Y. spinapophysis*, could also be presented in *Y. orthomargina*, but not clear show); spermathecae without distinct broader, touched or separated each other; fertilization ducts lamellar, appear in anterior-most edges of spermathecae.

###### Composition.

The genus currently includes 78 species.

*Yaginumaella aishwaryi* Sunil Jose, 2013, *Y. angulata* (Yang & Peng, 2023), comb. nov., *Y. armata* (Jastrzebski, 2011), *Y. badongensis* Song & Chai, 1992, comb. rest., *Y. bhutanica* Żabka, 1981, comb. rest., *Y. bulbosa* Peng, Tang & Li, 2008, comb. rest., *Y. cambridgei* Żabka, 1981, comb. rest., *Y. circula* (Yang & Peng, 2023), comb. nov., *Y. curvata* Li, Liu & Peng, 2024, *Y. dali* Shao, Li & Yang, 2014, *Y. danzhu* (Yang & Peng, 2023), comb. nov., *Y. daolangi* sp. nov., *Y. davidi* (Yang & Peng, 2023), comb. nov., *Y. daweishan* Wang, Mi, Li & Xu, 2024, *Y. erlang* Wang, Mi & Li, 2024, *Y. falcata* Zhu, Zhang, Zhang & Chen, 2005, comb. rest., *Y. filiforma* (Yang & Peng, 2023), comb. nov., *Y. flexa* Song & Chai, 1992, *Y. foliolata* (Yang & Peng, 2023), comb. nov., *Y. gemina* (Yang & Peng, 2023), comb. nov., *Y. gogonaica* Żabka, 1981, comb. rest., *Y. hagiang* Wang, Li & Pham, 2023, *Y. helvetorum* Żabka, 1981, comb. rest., *Y. hubeiensis* Li, Wang, Irfan & Peng, 2018, comb. rest., *Y. hybrida* Żabka, 1981, comb. rest., *Y. hyogoensis* Bohdanowicz & Prószyński, 1987, *Y. intermedia* Żabka, 1981, comb. rest., *Y. jietouensis* (Yang & Peng, 2023), comb. nov., *Y. linzhiensis* (Hu, 2001), comb. nov., *Y. longapophysis* (Yang & Peng, 2023), comb. nov., *Y. longlingensis* (Yang & Peng, 2023), comb. nov., *Y. longnanensis* Yang, Tang & Kim, 1997, *Y. lushiensis* Zhang & Zhu, 2007 comb. rest., *Y. lushuiensis* Liu, Yang & Peng, 2016, *Y. medog* sp. nov., *Y. medvedevi* Prószyński, 1979, *Y. moinba* Wang, Mi, Li & Xu, 2024, *Y. montana* Żabka, 1981, comb. rest., *Y. nepalica* Żabka, 1980, comb. rest. *Y. nobilis* Żabka, 1981, comb. rest., *Y. nova* Żabka, 1981, comb. rest., *Y. orientalis* Żabka, 1981, comb. rest., *Y. originalis* Żabka, 1981, comb. rest., *Y. orthomargina* Shao, Li & Yang, 2014, *Y. pentamaculata* (Hu, 2001), comb. nov., *Y. pilosa* Żabka, 1981, comb. rest., *Y. pingbian* Wang, Mi, Li & Xu, 2024, *Y. pseudoflexa* Liu, Yang & Peng, 2016, comb. rest., *Y. pulchella* Li, Wang, Irfan & Peng, 2018, comb. rest., *Y. qianlei* sp. nov., *Y. rectangula* (Yang & Peng, 2023), comb. nov., *Y. robusta* (Yang & Peng, 2023), comb. nov., *Y. senchalensis* Prószyński, 1992 , comb. rest., *Y. silvatica* Żabka, 1981, comb. rest., *Y. simoni* Żabka, 1981, comb. rest., *Y. songi* (Logunov, 1995), comb. nov., *Y. spinapophysis* sp. nov., *Y. stemmleri* Żabka, 1981, comb. rest., *Y. strandi* Żabka, 1981, comb. rest., *Y. striatipes* (Grube, 1861), *Y. subhubeiensis* (Wang, Mi & Peng, 2023), comb. nov., *Y. supina* Żabka, 1981, comb. rest., *Y. tenella* Żabka, 1981, comb. rest., *Y. tengchongensis* (Yang & Peng, 2023), comb. nov., *Y. tenzingi* Żabka, 1980, comb. rest., *Y. thakkholaica* Żabka, 1980, comb. rest., *Y. thimphuica* Żabka, 1981, comb. rest., *Y. umbellulata* (Yang & Peng, 2023), comb. nov., *Y. urbanii* Żabka, 1981, comb. rest., *Y. ususudi* (Yaginuma, 1972), *Y. variegata* (Logunov, 1995), comb. nov., *Y. versicolor* Żabka, 1981, comb. rest., *Y. wangdica* Żabka, 1981, comb. rest., *Y. wenxianensis* (Tang & Yang, 1995), *Y. wuermli* Żabka, 1981, comb. rest., *Y. xiaoqingi* sp. nov., *Y. zabkai* Wang, Mi & Peng, 2023, *Y. zonata* (Yang & Peng, 2023), comb. nov.

###### Distribution.

Bhutan, China, Japan, India, Kazakhstan, Korea, Myanmar, Nepal, Russia (Far East), Vietnam.

###### Comments.

54 species are transferred into the genus from *Ptocasius* and *Menemerus* because they are morphologically consistent with the type species and the related congeners. However, some species are uncertain, such as *Y. pilosa*, and *Y. stemmleri*, which present a different male palp from that of *Yaginumaella*, indicating they could not be actual members. They are also being transferred because they should have a similar habitus with *Yaginumaella* rather than *Ptocasius* (although their habitus has not been shown, a typical pattern of the described species in Żabka, 1981 was provided (Żabka 1981: fig. 3), which is consistent with *Yaginumaella*) and thus their generic position also needs further confirmation. The generic position of some remainders in *Ptocasius*, also requires additional attention. For example, *P. linzhiensis* (Hu, 2001) (transferred from *Morgus*) shares similar copulatory organs with the type species, but the habitus information is lacking, making the generic position cannot be confirmed appropriately; *P. dian* Wang, Mi & Peng, 2023 and *P. vittatus* Song, 1991 present a similar habitus pattern to the type species, but different in epigynal structure. Moreover, species described by Żabka (1981) and Yang and Peng (2023) are mostly known only from single females, and some of them share very similar epigynal structures, indicating the presence of potential synonyms and the need for further taxonomic revisions. *Ptocasius zabkai* Yang & Peng, 2023 and *Y. zonata* were collected in the sites close to each other and share indistinguishable epigynal structures and thus are considered conspecific. We act as First Revisor per ICZN (1999) and assign the former as a synonym of the latter. *Thyene incognita* (Żabka, 1981), comb. nov. is proposed because the path of the copulatory duct is almost identical to some of the Asian *Thyene* species. *Pancorius lobatus* (Peng, Tso & Li, 2002), comb. nov. (transferred from *Yaginumaella*) is proposed because it is closely similar to *P. submontanus* Prószyński, 1992, and it could be a junior synonym of the latter.

##### ﻿ Yaginumaella daolangi

Taxon classificationAnimaliaAraneaeSalticidae

C. Wang, Mi & Li sp. nov.

BAF52F2D-8F9E-507A-9347-E5037A6D5F00

https://zoobank.org/644AD353-6887-4799-AB25-D2A97691F99F

Figs 7, 8

###### Type material.

***Holotype*** • ♂ (TRU-JS 0839), China: Yunnan: Kunming City, Guandu District, Shajin Village (25°5.09'N, 103°1.02'E, ca 2,340 m), 20.x.2023; H. Qiu leg. ***Paratypes*** • 1♀ (TRU-JS 0840), same data as for holotype; • 1♂ (IZCAS-Ar45884), Sichuan, Miyi County, Binggu Township, Maidichong Village (26°40.76'N, 102°3.75'E, ca 2,240 m), 7.vi.2024, X.Q. Zhang, Y. Wang, and Q.Z. Meng leg.

###### Diagnosis.

The male of *Yaginumaella daolangi* sp. nov. resembles that of *Y. ususudi* (Yaginuma, 1972) in having a thick embolus originating from ca 9 o′clock position, but can be easily distinguished by the retrolateral tibial apophysis is curved towards retrolateral side distally (Fig. 7B, C) vs almost straight in *Y. ususudi* (Yaginuma 1972: fig. 10). The female of this new species can be easily distinguished from other congeners by postero-ventrally opened epigynal hood (Fig. 8A) vs epigynal hood posteriorly or posterolaterally opened in others (see the drawings of the congeners on Metzner 2025).

###### Description.

**Male** (Figs 7A–C, 8C, D, F, G). Total length 4.65. Cephalothorax 2.23 long, 1.68 wide. Abdomen 2.40 long, 1.39 wide. Eye sizes and inter distances: AME 0.43, ALE 0.26, PLE 0.24, AERW 1.45, PERW 1.39, EFL 0.90. Legs: I 4.61 (1.43, 0.85, 1.10, 0.75, 0.48), II 3.94 (1.25, 0.73, 0.85, 0.63, 0.48), III 4.57 (1.48, 0.68, 0.93, 0.93, 0.55), IV 4.87 (1.48, 0.68, 1.08, 1.08, 0.55). Carapace elevated, orange to dark, covered with sparse, long, dark setae on cephalic region, dense white setae behind PMEs, with pair of lateral submarginal white setal bands, and longitudinal, central yellow stripe extending across thorax; fovea red-brown, longitudinal. Chelicerae orange-yellow, with type dentition. Endites yellow with pale disto-inner portions, covered with clusters of dense dark setae on inner distal margins. Labium slightly darker than endites. Sternum yellow to brown, with truncated anterior edge and tapered distal portion, ~ 1.3× longer than wide. Legs setose, spiny. Dorsum of abdomen mainly dark and spotted, covered with sparse, long, dark setae and dense, shorter, dark and pale setae, with central, gradually broadened, longitudinal yellow stripe followed by transverse, sub-triangular yellow patch; venter colored as dorsum, with dotted lines.

Palp (Fig. 7A–C): tibia slightly wider than long in ventral view; retrolateral tibial apophysis strongly sclerotized, straight extending until curved retrolaterally at distal 1/3, with rather pointed tip; cymbium somewhat longer than wide, with truncated anterior edge; tegulum elongate-oval, with postero-prolaterally extended posterior lobe; embolus originates from ca 9 o′clock position, curved into almost C-shaped, abruptly narrowed and tapered at distal 1/4, with pointed tip directed towards ca 1:30 o′clock position.

**Female** (Fig. 8A, B, E). Total length 4.87. Cephalothorax 2.24 long, 1.69 wide. Abdomen 2.65 long, 1.69 wide. Eye sizes and inter distances: AME 0.44, ALE 0.26, PLE 0.24, AERW 1.46, PERW 1.46, EFL 0.96. Legs: I 4.01 (1.23, 0.80, 0.90, 0.63, 0.45), II 3.81 (1.18, 0.75, 0.85, 0.60, 0.43), III 4.50 (1.50, 0.70, 0.95, 0.85, 0.50), IV 4.91 (1.50, 0.68, 1.13, 1.10, 0.50). Habitus (Fig. 8E) similar to that of male.

Epigyne (Fig. 8A, B): longer than wide, with pair of mediolateral hoods opened posteroventrally; atrium almost inverted trapeziform, ~1/2 epigynal length; copulatory openings inclined, slit-shaped; copulatory ducts run posteriorly with curves until slightly enlarged and forming folds at posterior portions, and then extend contrary with curves to connect to spermathecae without distinct borders; fertilization ducts extending almost transversely.

###### Distribution.

China (Sichuan, Yunnan) (Fig. 2).

###### Etymology.

The specific name is after the famous Chinese singer, Daolang (real name Lin Luo); noun (name) in genitive case.

##### ﻿ Yaginumaella medog

Taxon classificationAnimaliaAraneaeSalticidae

C. Wang, Mi & Li sp. nov.

3467CFBB-A24A-562F-9985-EE52F4D1431C

https://zoobank.org/CBFCF461-C9EE-49D7-B13F-8A25EDF88C92

Figs 9, 10

###### Type material.

***Holotype*** • ♂ (TRU-JS 0841), China: Xizang Autonomous Region: Medog County, around Renqingbeng Temple (29°18.31'N, 95°21.29'E, ca 1,970 m), 26.v.2024, X.Q. Mi et al. Leg. ***Paratypes*** • 2♂2♀ (TRU-JS 0842–0845), same data as for holotype.

###### Diagnosis.

The male of *Yaginumaella medog* sp. nov. is unique for having two retromarginal cheliceral teeth. It somewhat resembles that of *Y. urbanii* Żabka, 1981, comb. rest. in having a very similar palpal structure, especially the origination of the embolus, but differs in: 1) the embolus is weakly sclerotized at the distal 1/3 (Fig. 9B) vs strongly sclerotized in *Y. urbanii* (Yu et al. 2024: fig. 6A); 2) the posterior tegular lobe is nearly round (Fig. 9B) vs sub-triangular in *Y. urbanii* (Yu et al. 2024: figs 6A, 7A). The female resembles that of *Y. nova* Żabka, 1981, comb. rest. in having a very similar epigynal structure, but differs in: 1) the distance between epigynal hoods is almost as wide as the atrium (Fig. 10A, B) vs ~ 1.2× wider than the atrium in *Y. nova* (Żabka 1981: fig. 44); 2) the copulatory ducts are enlarged into balls medially (Fig. 10A, B) vs not enlarged in *Y. nova* (Żabka 1981: figs 44, 45).

###### Description.

**Male** (Figs 9A–C, 10E, G–I). Total length 4.48. Cephalothorax 2.27 long, 1.88 wide. Abdomen 2.23 long, 1.36 wide. Eye sizes and inter distances: AME 0.49, ALE 0.28, PLE 0.25, AERW 1.58, PERW 1.52, EFL 1.06. Legs: I 5.62 (1.63, 0.95, 1.43, 0.98, 0.63), II 4.59 (1.45, 0.75, 1.13, 0.78, 0.48), III 5.41 (1.63, 0.78, 1.25, 1.20, 0.55), IV 5.54 (1.63, 0.78, 1.28, 1.30, 0.55). Carapace mainly dark brown, covered with dense dark and golden setae, with pair of lateral submarginal pale setal bands; fovea dark red, linear. Chelicerae with three promarginal and two retromarginal teeth. Endites pale, with cluster of dense dark setae on disto-inner portions. Labium dark brown except distal portion pale. Sternum dark brown, ~1.2× longer than wide. Legs pale to brown, spiny. Dorsum of abdomen with pale longitudinal patch distally across with much thinner, transverse pale stripe; venter mainly pale, with central, longitudinal, dark patch and irregular dark spots.

Palp (Fig. 9A–C): tibia ~ 1.56× longer than wide in ventral view, with straight retrolateral apophysis tapered to pointed tip directed upward in retrolateral view; cymbium ~1.43× longer than wide, setose; tegulum flat, with almost round posterior lobe; embolus originates from ca 9 o′clock position, curved ~1/4 circle, distal 1/3 weakly sclerotized.

**Female** (Fig. 10A–D, F). Total length 4.58. Cephalothorax 2.00 long, 1.61 wide. Abdomen 2.52 long, 1.73 wide. Eye sizes and inter distances: AME 0.48, ALE 0.28, PLE 0.25, AERW 1.55, PERW 1.48, EFL 1.06. Legs: I 3.83 (1.20, 0.70, 0.85, 0.63, 0.45), II 3.63 (1.10, 0.70, 0.80, 0.60, 0.43), III 4.54 (1.33, 0.73, 0.95, 0.98, 0.55), IV 4.84 (1.38, 0.70, 1.08, 1.13, 0.55). Habitus (Fig. 10F) similar to that of male except with pale, longitudinal, thoracal stripe and only with two promarginal cheliceral teeth and one retromarginal tooth.

Epigyne (Fig. 10A–D): with pair of almost bell-shaped hoods posterior to base of copulatory openings; atrium nearly oval, anteromedially located; copulatory openings slit-shaped, beneath posterolateral portion of atrium; copulatory ducts curved into almost C-shape at origin, and then slightly enlarged into balls and continue to connect to elongate-oval spermathecae; fertilization ducts originate from anterior-most edges of spermathecae.

###### Distribution.

Know only from the type locality in Xizang, China (Fig. 2).

###### Etymology.

The specific name is after the type locality , Medog County; noun in apposition.

##### ﻿ Yaginumaella qianlei

Taxon classificationAnimaliaAraneaeSalticidae

C. Wang, Mi & Li sp. nov.

C8681AAD-D2F6-5C52-A0B3-D810B1C34E43

https://zoobank.org/60F8A1DD-1BAA-4287-986A-5E91B23C0881

Figs 11, 12

###### Type material.

***Holotype*** • ♂ (TRU-JS 0846), China: Sichuan: Jiulong County, Wuxuhai (29°6.91'N, 101°24.04'E, ca 3,470 m), 1.ix.2020, Q.L. Lu leg. ***Paratypes*** • 1♂1♀ (TRU-JS 0847–0848), same data as for holotype.

###### Diagnosis.

The male of *Yaginumaella qianlei* sp. nov. is unique for having a triangular process medially on the retrolateral cymbial margin. It somewhat resembles that of *Y. pulchella* Li, Wang, Irfan & Peng, 2018, comb. rest. in having a similar palpal structure, but it differs in: 1) the tegulum is almost 1.16× longer than wide in ventral view (Fig. 11B) vs ~1.5× longer than wide in *Y. pulchella* (Li et al. 2018: figs 1B, 2A); 2) the retrolateral tibial apophysis is slightly curved inward in retrolateral view (Fig. 11C) vs straight in *Y. pulchella* (Li et al. 2018: figs 1C, 2B). The female of this new species resembles that of *Y. medvedevi* Prószyński, 1979 in having broad epigynal hoods, but can be easily distinguished by the epigynal hoods are far away from the atrium (Fig. 12A) vs touching the lowest margin of the atrium in *Y. medvedevi* (Prószyński 1979: fig. 318).

###### Description.

**Male** (Figs 11A–C, 12C, E–G). Total length 4.12. Cephalothorax 1.81 long, 1.41 wide. Abdomen 2.18 long, 1.52 wide. Eye sizes and inter distances: AME 0.36, ALE 0.23, PLE 0.21, AERW 1.14, PERW 1.09, EFL 0.74. Legs: I 3.46 (1.00, 0.63, 0.80, 0.55, 0.48), II 2.99 (0.95, 0.53, 0.63, 0.50, 0.38), III 3.18 (1.00, 0.45, 0.63, 0.65, 0.45), IV (1.10, 0.50, 0.75, missing, missing). Carapace mainly dark, covered with dark golden and pale setae of various lengths, with longitudinal, central, pale, gradually narrowed stripe extending from anterior area of fovea to distal end; fovea dark red. Chelicerae with typical dentition. Endites with disto-inner portions and bearing dense dark setae on disto-inner margins. Labium tapered from base, with pale distal end. Sternum mainly green-brown except with centrally pale yellow, with straight anterior edge. Legs pale to red brown, with dark irregular patches. Dorsum of abdomen dark laterally and setose, with central, irregular pale patch; venter mainly pale, with dark dots and central, longitudinal, patch extending across whole surface.

Palp (Fig. 11A–C): tibia slightly wider than long, with strongly sclerotized, tapered retrolateral apophysis slightly curved inward distally and pointed apically; cymbium somewhat longer than wide, with sub-triangular process located on median portion of retrolateral side; tegulum swollen medioposteriorly, with blunt posterior lobe; embolus arises at ca 9 o′clock position, curved at proximal and then antero-retrolaterally extending to rather pointed tip directed towards ca 1:30 o′clock position.

**Female** (Fig. 12A, B, D). Total length 4.37. Cephalothorax 1.79 long, 1.40 wide. Abdomen 2.46 long, 1.69 wide. Eye sizes and inter distances: AME 0.36, ALE 0.23, PLE 0.21, AERW 1.14, PERW 1.17, EFL 0.80. Legs: I 2.89 (0.88, 0.50, 0.63, 0.45, 0.43), II 2.71 (0.85, 0.48, 0.60, 0.40, 0.38), III 3.21 (1.00, 0.50, 0.65, 0.63, 0.43), IV 3.82 (1.13, 0.55, 0.88, 0.83, 0.43). Habitus (Fig. 12D) similar to that of male except pale in color, and longitudinal thoracic stripe much broader.

Epigyne (Fig. 12A, B): slightly wider than long, with pair of mediolateral hoods; copulatory openings slit-shaped, anteriorly located; copulatory ducts curved into C-shapes at origin, and then form folds and connect to spermathecae without distinct broader; fertilization ducts appear from anterior-most edges of spermathecae.

###### Distribution.

Known only from the type locality in Sichuan, China (Fig. 2).

###### Etymology.

The specific name is after the collector, Mr Qianle Lu; noun (name) in genitive case.

##### ﻿ Yaginumaella spinapophysis

Taxon classificationAnimaliaAraneaeSalticidae

C. Wang, Mi & Li sp. nov.

D9987D3C-FC57-5818-B10C-151754271275

https://zoobank.org/F84EDD0E-0E57-45E6-B331-F810E0114C92

Figs 13, 14

###### Type material.

***Holotype*** • ♂ (IZCAS-Ar45885), China: Sichuan: Miyi County, Binggu Township, Maidichong Village (26°40.76'N, 102°3.75'E, ca 2,240 m), 7.vi.2024, X.Q. Zhang, Y. Wang, and Q.Z. Meng leg. ***Paratypes*** • 3♀ (IZCAS-Ar45886–45888), same data as for holotype.

###### Diagnosis.

The male of *Yaginumaella spinapophysis* sp. nov. can be easily distinguished from congeners except *Y. orthomargina* Shao, Li & Yang, 2014 and *Y. hagiang* Wang, Li & Pham, 2023 by the presence of embolic division, but can be easily distinguished from those of two species by the bifurcated RTA with spinous ventral ramus (Fig. 13C) vs non-bifurcated in both of those species (Shao et al. 2014: fig. 11; Wang et al. 2023a: fig. 22B). The female of this new species resembles that of *Y. orthomargina* in general shape of epigyne, especially the anteriorly located, single atrium, but differs by the almost round atrium (Fig. 14A–D) vs elongate-oval in *Y. orthomargina* (Shao et al. 2014: figs 8, 9).

###### Description.

**Male** (Figs 13A–C, 14E, F, H, I). Total length 5.89. Cephalothorax 2.83 long, 2.23 wide. Abdomen 3.25 long, 2.11 wide. Eye sizes and inter distances: AME 0.53, ALE 0.30, PLE 0.26, AERW 1.74, PERW 1.66, EFL 1.17. Legs: I 6.36 (1.83, 1.03, 1.60, 1.20, 0.70), II 5.42 (1.63, 0.98, 1.28, 0.93, 0.60), III 6.03 (1.75, 0.88, 1.40, 1.35, 0.65), IV 6.48 (1.88, 0.90, 1.50, 1.55, 0.65). Carapace elevated, mainly red-brown, with paler area anteriorly on central portion of thorax, clusters of white setae on anterolateral portion of eye field and posterior portion of thorax, pair of white lateral sub-marginal setal bands extending from spaces between ALEs and AMEs to posterior end, and dense dark setae on lateral sides of thorax and near AMEs; fovea dark red, line-shaped. Chelicerae red-brown, with typical dentition. Endites broadened distally, with dense disto-inner marginal dark setae. Labium darker than endites. Sternum dark brown, with two sub-round spots. Legs spiny. Dorsum of abdomen mainly dark, spotted, with longitudinal, central dark yellow patch followed by pair of oblique yellow stripes bearing white setae, and several transverse, arc-shaped dotted lines; venter mainly dark, with pair of longitudinal median dotted lines with slight curves.

Palp (Fig. 13A–C): tibia almost as long as wide in retrolateral view; retrolateral tibial apophysis bifurcated with spinous ventral ramus and flat, sheet-shaped dorsal ramus; cymbium setose, with truncated anterior edge; tegulum elongate-oval, with posteriorly extended posterior lobe; embolus strongly sclerotized, arises at ca 9 o′clock position, runs along tegular margin at origin, and then antero-retrolaterally extended to pointed tip, basally fused with twisted division with blunt terminus.

**Female** (Fig. 14A–D, G). Total length 5.88. Cephalothorax 2.39 long, 1.76 wide. Abdomen 3.53 long, 2.83 wide. Eye sizes and inter distances: AME 0.50, ALE 0.29, PLE 0.26, AERW 1.54, PERW 1.51, EFL 1.10. Legs: I 4.04 (1.33, 0.80, 0.73, 0.68, 0.50), II 4.04 (1.28, 0.75, 0.88, 0.63, 0.50), III 4.83 (1.45, 0.75, 1.03, 1.00, 0.60), IV 5.34 (1.58, 0.78, 1.18, 1.20, 0.60). Habitus (Fig. 14G) similar to that of male except lacking pair of white lateral sub-marginal setal bands and with dense white setae centrally on thorax.

Epigyne (Fig. 14A–D): slightly longer than wide, with pair of posteriorly extended hoods ~1.5× longer than wide and lateral to basal portion of atrium; atrium anteriorly located, almost round; copulatory ducts forming complicated coils, anteromedially attached straight, bar-shaped, anterolaterally extended secondary spermathecae/accessory glands; spermathecae without distinct border, distinct from each other ~2/3 atrial width; fertilization ducts originate from anterior-most edges of spermathecae.

###### Distribution.

Known only from the type locality in Sichuan, China (Fig. 2).

###### Etymology.

The specific name is a combination of spin (refers to spinosus) and apophysis, which refers to the spiniform ventral ramus of retrolateral tibial apophysis; noun in apposition.

##### ﻿ Yaginumaella xiaoqingi

Taxon classificationAnimaliaAraneaeSalticidae

C. Wang, Mi & Li sp. nov.

F616FB63-CA7E-5836-B464-1352E951CB0E

https://zoobank.org/85A9181E-7574-4303-99E6-2FB4470B82DD

Figs 15, 16

###### Type material.

***Holotype*** • ♂ (IZCAS-Ar45889), China: Guizhou: Weining Yi, Hui and Miao Autonomous County, Guanfenghai Township, Shazipo Village (26°55.68'N, 103°58.29'E, ca 2,250 m), 1.vi.2024, X.Q. Zhang, Y. Wang, and Q.Z. Meng leg. ***Paratypes*** • 2♀ (IZCAS-Ar45890–45891), same data as for holotype; • 1♂1♀ (IZCAS-Ar45892–45893), Shuijinbaobao Village (26°53.52'N, 104°5.72'E, ca 2,530 m), same data and collectors as holotype.

###### Diagnosis.

*Yaginumaella xiaoqingi* sp. nov. closely resembles *Y. erlang* Wang, Mi & Li, 2024 in having very similar copulatory organs, but can be distinguished by the following: 1) the retrolateral tibial apophysis has blunt tip in retrolateral view (Fig. 15B) vs pointed in *Y. erlang* (Wang et al. 2024a: fig. 20C); 2) the posterior tegular lobe extends postero-retrolaterally (Fig. 15C) vs posteriorly in *Y. erlang* (Wang et al. 2024a: fig. 20B); 3) the atrium is inverted trapeziform (Fig. 16A) vs trapeziform in *Y. erlang* (Wang et al. 2024a: fig. 21A).

###### Description.

**Male** (Figs 15A–D, 16C, D, F, G). Total length 4.15. Cephalothorax 2.05 long, 1.49 wide. Abdomen 2.23 long, 1.51 wide. Eye sizes and inter distances: AME 0.38, ALE 0.23, PLE 0.21, AERW 1.24, PERW 1.21, EFL 0.77. Legs: I 3.96 (1.18, 0.70, 0.93, 0.70, 0.45), II 3.49 (1.05, 0.65, 0.78, 0.58, 0.43), III 3.80 (1.20, 0.60, 0.75, 0.80, 0.45), IV 4.18 (1.25, 0.58, 0.90, 0.95, 0.50). Carapace yellow-brown to brown, covered with dense pale yellow and sparse dark setae, with longitudinal, central pale yellow stripe extending from posterior portion of eye field to posterior end; fovea linear. Chelicerae yellow, bearing long dark setae on anterior surface, with type dentition. Endites pale yellow, with pale disto-inner portions and dense disto-inner marginal setae. Labium almost as color as endites. Sternum pale yellow, almost shield-shaped. Legs pale to yellow-brown, spiny. Dorsum of abdomen covered with lateral dark and pale setae, with central, irregular pale yellow patch and two pairs of muscle depressions; venter mainly pale, with dark dots.

Palp (Fig. 15A–D): tibia slightly wider than long in retrolateral view; retrolateral tibial apophysis strongly sclerotized, tapered, ca 1.5× longer than tibia, slightly curved medially and with blunt tip; cymbium somewhat longer than wide; tegulum almost oval, swollen medio-posteriorly, with posterior lobe extending retrolatero-posteriorly; embolus originates from ca 8 o′clock position, tapered, curved ~ 1/4 circle and with rather pointed tip.

**Female** (Fig. 16A, B, E). Total length 4.36. Cephalothorax 2.11 long, 1.67 wide. Abdomen 2.36 long, 1.61 wide. Eye sizes and inter distances: AME 0.40, ALE 0.26, PLE 0.23, AERW 1.36, PERW 1.33, EFL 0.89. Legs: I 3.75 (1.15, 0.70, 0.85, 0.60, 0.45), II 3.46 (1.10, 0.65, 0.78, 0.50, 0.43), III 3.94 (1.20, 0.63, 0.80, 0.83, 0.48), IV 4.57 (1.38, 0.63, 1.00, 1.05, 0.51). Habitus (Fig. 16E) similar to that of male except darker.

Epigyne (Fig. 16A, B): almost as long as wide, with pair of sub-triangular anterolateral hoods lateral to anterior portion of atrium; atrium anteriorly located, inverted trapeziform; copulatory openings slit-shaped; copulatory ducts curved into C-shapes at origin and followed by complicated coils; spermathecae without distinct border; fertilization ducts transversely extending.

###### Distribution.

Known only from the type locality in Guizhou, China (Fig. 2).

###### Etymology.

The specific name refers to Dr Xiaoqing Zhang, one of the collectors; noun (name) in genitive case.

## ﻿Discussion

Counting the present work, the current number of Chinese species of Plexippina has increased to 154 species in 19 genera, which is approximately 25% of the known worldwide species members of the subtribe (WSC 2025). However, it is probable that the diversity of Chinese Plexippina will continue to rapidly increase because most of the region still remains poorly surveyed. According to the current species distribution and our experience, predictably, most of the undiscovered species will be found in the southwest mountainous and Himalayan regions, where there are two diversity centers of the subtribe.

In the ML tree, the general relationships within Plexippina are mainly consistent with previous studies, such as Kanesharatnam and Benjamin (2021) and Marathe et al. (2024). For example, *Colopsus* Simon, 1902, is closely related to *Pancorius* Simon, 1902, *Tenkana* Marathe, Maddison & Caleb, 2024 is related to *Hyllus* C.L. Koch, 1846. However, there are some exceptions, such as the generic position of *Telamonia* Thorell, 1887 that is sister to *Ghatippus* Marathe & Maddison, 2024. In the new ML tree, differing from Marathe et al. (2024), whose results strongly support *Telamonia* as closely related to *Tenkana* and *Hyllus*. This inconsistency could be related to the different species selected for Plexippina, or a lack of sufficient molecular information in our study.

Although the phylogenetic analysis reveals *Ptocasius* and *Yaginumaella* are separated genera, and more than 50 new and restored combinations are also proposed, the generic position of several members placed in these two genera remains uncertain due to a lack of sufficient morphological or molecular evidence. So, further taxonomic attention on these two genera is also necessary. Within *Yaginumaella*, *Y. spinapophysis* sp. nov. and its congeners, *Y. orthomargina* Shao, Li & Yang, 2014 and *Y. hagiang* Wang, Li & Pham, 2023 are very specific in having the embolic division and remarkable secondary spermatheca/accessory gland. In the ML tree, *Y. spinapophysis* sp. nov. is also sister to other species. Based on the above, those three species can be further divided into a species group at least, or an even better choice is to split them from *Yaginumaella*, especially if more similar species are discovered, which will be helpful for rapid recognition of the highly diverse *Yaginumaella* and related species, but molecular studies of those species should provide a solution.

## Supplementary Material

XML Treatment for Chuanattus

XML Treatment for Chuanattus deelemanae

XML Treatment for Dianattus

XML Treatment for Dianattus proszynskii

XML Treatment for Yaginumaella

XML Treatment for Yaginumaella daolangi

XML Treatment for Yaginumaella medog

XML Treatment for Yaginumaella qianlei

XML Treatment for Yaginumaella spinapophysis

XML Treatment for Yaginumaella xiaoqingi

## ﻿Additional information

### Use of AI

No use of AI was reported.

### Author ORCIDs

Cheng Wang https://orcid.org/0000-0003-1831-0579

Ying Wang https://orcid.org/0009-0007-9173-4438

Xiaoqi Mi https://orcid.org/0000-0003-1744-3855

Shuqiang Li https://orcid.org/0000-0002-3290-5416

### Data availability

All of the data that support the findings of this study are available in the main text.
